# The NSAID glafenine rescues class 2 CFTR mutants via cyclooxygenase 2 inhibition of the arachidonic acid pathway

**DOI:** 10.1038/s41598-022-08661-8

**Published:** 2022-03-17

**Authors:** Graeme W. Carlile, Qi Yang, Elizabeth Matthes, Jie Liao, Véronique Birault, Helen F. Sneddon, Darren L. Poole, Callum J. Hall, John W. Hanrahan, David Y. Thomas

**Affiliations:** 1 Department of Biochemistry, Cystic Fibrosis Translational Research Centre, McGill University, McIntyre Medical Sciences Building, 3655 Promenade Sir William Osler, Montreal, QC H3G 1Y6 Canada; 2 Department of Human Genetics, Cystic Fibrosis Translational Research Centre, McGill University, Montreal, QC H3G 1Y6 Canada; 3 Department of Physiology, McGill Cystic Fibrosis Translational Research Centre, McGill University, Montreal, QC H3G 1Y6 Canada; 4 Translation Department, The Francis Crick Institute, 1 Midland Road, London, NW1 1AT UK; 5 Department of Chemistry, Green Chemistry Centre of Excellence, University of York, Heslington, York, YO10 5DD UK; 6 Medicinal Chemistry, GlaxoSmithKline, Gunnels Wood Road, Stevenage, SG1 2NY UK

**Keywords:** Biochemistry, Drug discovery, Physiology

## Abstract

Most cases of cystic fibrosis (CF) are caused by class 2 mutations in the cystic fibrosis transmembrane regulator (CFTR). These proteins preserve some channel function but are retained in the endoplasmic reticulum (ER). Partial rescue of the most common CFTR class 2 mutant, F508del-CFTR, has been achieved through the development of pharmacological chaperones (Tezacaftor and Elexacaftor) that bind CFTR directly. However, it is not clear whether these drugs will rescue all class 2 CFTR mutants to a medically relevant level. We have previously shown that the nonsteroidal anti-inflammatory drug (NSAID) ibuprofen can correct F508del-CFTR trafficking. Here, we utilized RNAi and pharmacological inhibitors to determine the mechanism of action of the NSAID glafenine. Using cellular thermal stability assays (CETSAs), we show that it is a proteostasis modulator. Using medicinal chemistry, we identified a derivative with a fourfold increase in CFTR corrector potency. Furthermore, we show that these novel arachidonic acid pathway inhibitors can rescue difficult-to-correct class 2 mutants, such as G85E-CFTR > 13%, that of non-CF cells in well-differentiated HBE cells. Thus, the results suggest that targeting the arachidonic acid pathway may be a profitable way of developing correctors of certain previously hard-to-correct class 2 CFTR mutations.

## Introduction

Cystic fibrosis (CF) is a severe, multiorgan orphan disease that affects over 70,000 people worldwide^1^. It is caused by mutations in the CF transmembrane conductance regulator gene (*CFTR*) that lead to CFTR protein defects^2,3^. CFTR is an anion channel that mediates cAMP-stimulated chloride and bicarbonate transport at mucosal surfaces^3,4^. Clinical manifestations of CF include chronic endobronchial infections, decreased lung function, exocrine pancreatic insufficiency, CF-related diabetes and reduced life expectancy^1,3,4^.

Approximately 90% of people with CF carry at least one copy of the class 2 CFTR mutation, *F508del*-*CFTR* (http://www.genet.sickkids.on.ca/cftr)^5,6^. Class 2 mutations cause CFTR misfolding, retention by the ER quality control (ERQC) mechanism and proteasomal degradation^7,8^. Low temperature (26–30 °C) partially restores F508delCFTR trafficking and channel function in cell lines, although the corrected channel has a lower open probability than wild-type CFTR^9^. A major focus of CF research has been to develop molecules that correct the folding and trafficking defects of the mutant protein^10,11^. There are two general mechanisms that drugs known as “correctors” can use to correct the mislocalization of F508del-CFTR and rescue F508del-CFTR function: (1) pharmacological chaperones, which bind directly to the misfolded protein to increase folding and ERQC escape, and (2) proteostasis modulators, which alter the folding and trafficking environment to favor mutant rescue^12^.

The clinically approved corrector drugs developed to date are pharmacological chaperones^13^, used along with drugs able to potentiate ion channel opening (potentiators) when CFTR is at the cell surface. The most recent clinical drug combination is Trikafta (Vertex Pharmaceuticals), a combination of correctors, Tezacaftor + Elexacaftor and the potentiator Ivacaftor. It is effective for patients with at least one F508del-CFTR copy and improves lung function by ~ 13.8%, as measured using forced expiratory volume in 1 s (FEV-1). Preliminary evidence further suggests that Trikafta will also be effective in correcting rarer class 2 CFTR mutations^13,14^. However, the correction level is variable and significantly below that attained for F508del and potentially below that needed for clinical benefit^13–15^. Also there are reports of significant liver damage in a small portion of patients receiving Trikafta^16,17^. Additionally, previous CFTR drug combination experience with Orkambi (which included the pharmacological chaperone Lumacaftor as well as the potentiator Ivacaftor) was that upon clinical use 25% of F508del-CFTR patients, and those with other class 2 mutations (e.g., N1303K and G85E) did not derive a benefit^18–21^ even though, like the F508del-CFTR mutant protein, both the proteins for N1303K and G85E are active if trafficked to the plasma membrane^13,14,22,23^. Taking all this into consideration along with the fact that over 2000 mutations have been identified in the *cftr* gene with an unknown number belonging to class 2, the need to expand the arsenal of CFTR corrector therapies to develop complete clinical coverage of class 2 CFTR mutation-derived CF becomes clear.

To find Class 2 CFTR mutant correctors that display a wider spectrum of correction capability, we turned to proteostasis modulators. We have previously described compounds as proteostasis modulators, in particular the NSAIDs ibuprofen and glafenine^24–27^. Some NSAIDS are widely used in the clinic, and some have proven to have liabilities. Glafenine has been clinically discontinued due to hepatoxicity^28^. It is used here as a probe of the correction pathway and as a proof of principle for this proteostatic approach to CF treatment.

We show here that like other NSAIDs, glafenine not only acts as a proteostasis modulator, it is one of the most potent NSAID correctors of F508del-CFTR and that its potency can be improved by medicinal chemistry: compound 49 gives a fourfold increase in F508del-CFTR correction over glafenine in human primary bronchial epithelial cells (HBE). We demonstrate that its target is cyclooxygenase 2 (COX2) and that its mutant CFTR corrector mechanism of action operates via the arachidonic acid pathway, preventing the conversion of arachidonic acid to prostaglandin E2 (PGE2). We also identified 2-(9-chloro-1H-phenanthro [9,10-d] imidazol-2-yl)-1,3-benzenedicarbonitrile (MF63) as a potent proteostatic regulator of F508del-CFTR. Furthermore, MF63 rescues class 2 CFTR mutations by preventing the stimulation of prostaglandin E_2_ receptor 4 (EP4). We also show that this CFTR class 2 mutant correction involved not only F508del-CFTR but also N1303K and G85E. Indeed, testing in HBE cells expressing G85E glafenine gave a correction level that was twice the response of lumacaftor. Compound 49 and MF63 gave significant correction equivalent to 9.5% (± 1.9%) and 13.4% (± 2.1%) of non-CF patients, respectively, which was significantly better than Trikafta (6.1% (± 1.1%) of non-CF patients) achieved in the same cell type. These results demonstrate that the exploration of proteostasis modulators is a productive strategy in the quest for CF therapeutics caused by rare class 2 CFTR mutations.

## Results

### NSAIDs correct F508del-CFTR trafficking

We have previously demonstrated that the NSAIDs ibuprofen and glafenine could correct F508del-CFTR mislocalization^24,26^. To determine whether this was a broad feature of NSAID biology, we tested 38 NSAIDs in the HTS F508del-CFTR cell surface expression assay (Suppl. Table 1). The results show that 7 of the 8 groups contained compounds that gave significant correction levels, the exception being the salicylate group. Glafenine, with 27.5% (± 1.1%) wild-type surface CFTR expression, gave the strongest corrector signal (n = 4).

### Glafenine does not bind F508del-CFTR

**Figure 1:**
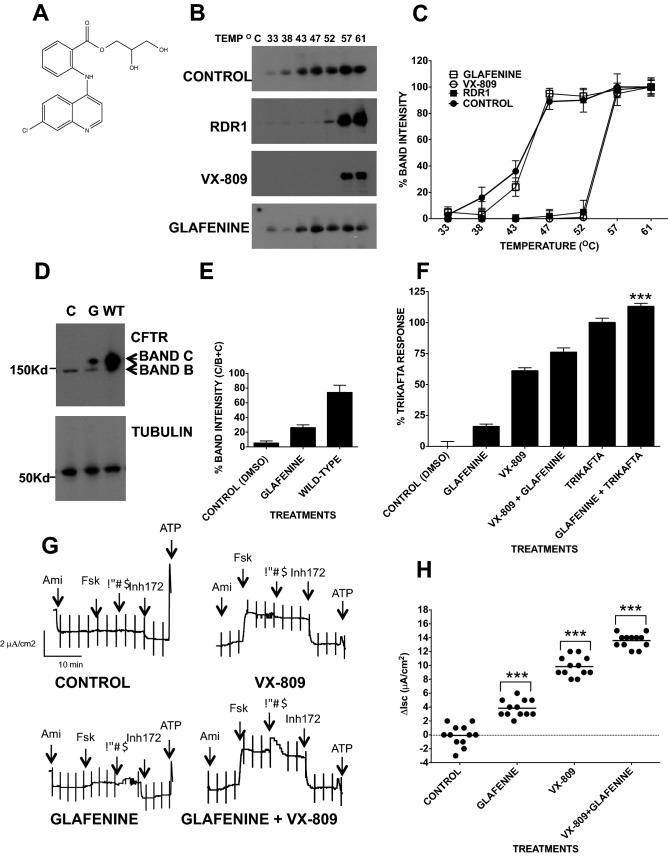
Protein trafficking and electrophysiological assays revealed correction of F508del-CFTR by glafenine. (**A**) A 2-dimensional chemical structure of glafenine (2,3-dihydroxypropyl 2-[(7-chloroquinolin-4-yl) amino] benzoate; hydrochloride). (**B**) CETSA assay showing representative immunoblots of the pellets after cell lysates were incubated for 10 min at different temperatures (33, 38, 43, 47, 52, 57 and 61 °C) in the presence of CFTR correctors. RDR1 and VX-809 are known pharmacological chaperones and were used here as positive controls. All correctors were tested at 10 μM except VX-809 (1 μM). Blots were probed with a monoclonal anti-CFTR antibody (n = 4). (**C**) Graph representing the band intensities for the immunoblots show in (**B**). (**D**) Immunoblot of F508del-CFTR expressed in BHK cells after 24 h of treatment with glafenine (10 μM) and with BHK cells expressing wild-type CFTR. (n = 4), (**E**) Relative intensity of bands B and band C in each lane in (**D**) as measured by ImageJ. (**F**) Cell-based HTS assay measuring surface F508del-CFTR in BHK cells after 24 h of treatment with glafenine at 10 μM and either VX-809 at 1 µM or Trikafta both separately and together with glafenine (n = 5). The asterisk in the graph represent a value significantly (level) above the results recorded for Trikafta alone (**G**). Representative *I*_*sc*_ response traces for F508del-CFTR functional expression in well-differentiated primary human bronchial epithelial (HBE) cells determined from the increase in short-circuit current stimulated by acute addition of forskolin + genistein (Δ*I*sc). The basolateral membrane was permeabilized using nystatin, and an apical-to-basolateral chloride gradient was imposed by sequential addition of 10 µM forskolin, 50 µM genistein, and 10 µM CFTRinh-172 after 24 h of preincubation with 0.1% dimethylsulfoxide (vehicle), glafenine (10 µM) or VX-809 (1 μM) individually and in combination (n = 4). (**H**) Graph for each compound for the data attained from the Ussing chamber (**G**). Data in (**F**) and (**H**) are presented as the means ± SEM, n = 4, *p < 0.05, **p < 0.01 and ***p < 0.001.

To determine whether glafenine directly interacts with F508del-CFTR, we used a cellular thermal shift assay (CETSA: Fig. 1B,C Suppl. Fig. 1)^29^. BHK cell lysates containing F508del-CFTR were divided into identical aliquots and treated for 10 min with 10 µM glafenine at temperatures between 33 and 61 °C. Lysates were then centrifuged to remove aggregates, and the pellets were run on polyacrylamide gels and immunoblotted for F508del-CFTR (n = 4).

The rationale is that as proteins are heated, they lose their folded structure, aggregate, become insoluble and are subsequently collected in the pellet. Compounds directly binding to CFTR (pharmacological chaperones) generally increase their thermostability, inhibiting the loss of fold. Cell lysis and the short test compound incubation period mean that the CFTR thermostability can only be altered by the compounds’ direct interaction with CFTR. RDR1 and VX-809 are both known pharmacological chaperones^29^, with RDR-1 showing a shift of 19 °C and VX-809 showing a Tm shift of 24 °C. In contrast, glafenine shows no shift in thermostability with both the control and the glafenine treated sample showing aggregation beginning at 33 °C (n = 4). This is further supported when one considers the band intensity measurements for these experiments that show that there is no significant difference between the control and the glafenine treated samples at any of the temperatures tested. Thus, when Fig. 1B,C are taken together this strongly suggesting that it does not interact directly with CFTR.

### Demonstration that glafenine is a proteostasis modulator

To further test this hypothesis that the NSAID glafenine (2-[(7-chloro-4-quinolinyl) amino] benzoic acid 2,3-dihydroxypropyl ester is a CFTR corrector^24^, we quantified the amount of mature complex glycosylated Band C F508del-CFTR protein that developed on western blot of cell lysates from BHK cells exposed to glafenine (10 μM) for 24 h. The principle of this approach is that the appearance of band C shows F508del-CFTR correctly trafficking out of the endoplasmic reticulum (ER) and to the Golgi apparatus where the final stages of glycosylation occur. No band C was detected under control conditions, but after 24 h of glafenine exposure, band C was detected (n = 4) (Fig. 1D,E Suppl. Fig. 2), with 24% (± 2.6%) of the F508del-CFTR signal in the glafenine-treated lane being band C compared with 72% (± 9.1%) for the wild-type CFTR lane. To measure F508del-CFTR surface expression rescued by either glafenine alone or in combination with either VX-809 or Trikafta we utilized the HTS assay (n = 5) (Fig. 1F). We measured surface F508del-CFTR after 24-h incubation with 10 μM glafenine alone and in combination with the pharmacological chaperone VX-809 (Lumacaftor 1 μM) or with Trikafta. All treatments significantly increased cell surface F508del-CFTR. Glafenine alone generated 14% (± 1.1%) surface expression of Trikafta whereas VX-809 gave 62% (± 1.9%) of the level generated by Trikafta, and in combination with VX-809, it gave additive correction of 75% (± 3%) of Trikafta. In combination with Trikafta, glafenine gave an additive response at 116% (± 2.4%) of TrIkafta alone. The additive effects that glafenine produces in concert with either VX-809 or Trikafta is consistent with its distinct mechanism and suggest a new therapeutic avenue for CF.

We also measured the maximal CFTR function in well-differentiated primary HBE cells expressing F508del-CFTR as the *I*_sc_ evoked by forskolin + genistein (n = 4) (Fig. 1G,H). Glafenine alone increased the forskolin + genistein response to 19.5% (± 0.2.2%) of those for VX-809, similar to the HTS assay (Fig. 1F). Exposure to glafenine + VX-809 yielded 120% (± 1.8%) of the response to VX-809 alone. Thus, glafenine partially corrects the function of endogenously expressed homozygous F508del-CFTR in well-differentiated patient-derived HBE cells. This functional response was sensitive to the CFTR-specific inhibitor CFTRinh-172, confirming that the responses were mediated by F508del-CFTR (Fig. 1G).

### Glafenine derivatization identifies a more potent F508del-CFTR corrector

**Figure 2:**
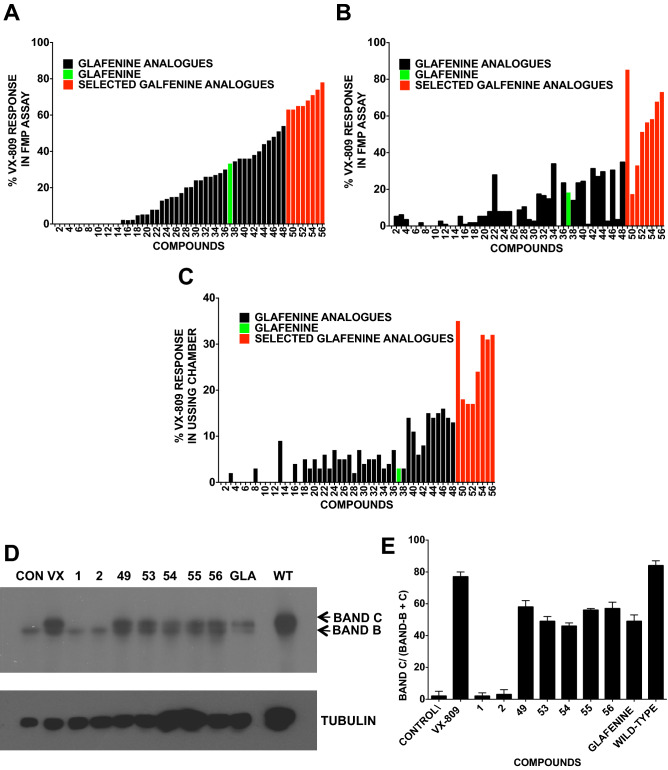
Cell-based and electrophysiological assays demonstrate the ability of glafenine derivatives to correct F508del-CFTR. (**A**) Cell-based HTS assay measuring surface F508del-CFTR in BHK cells after 24 h of treatment with glafenine and its analogs at 10 μM. Glafenine (compound 37) is marked in green, most analogs in black and the best responding analogs in red (n = 4). (**B**) FMP assay (FMP) that monitors membrane depolarization induced by forskolin + genistein when cells are pretreated for 24 h with glafenine and its derivatives (10 μM) performed in BHK cells expressing F508del-CFTR (n = 4). (**C**) F508del-CFTR functional expression in well-differentiated CFBE41o- cell epithelial cells determined from the increase in *Isc* stimulated by acute addition of forskolin + genistein (Δ*I*sc). The basolateral membrane was permeabilized using nystatin, and an apical-to-basolateral chloride gradient was imposed. All compounds tested at 10 µM for 24 h (n = 4). (**D**) Immunoblot of F508del-CFTR in BHK cells after 24 h of treatment with VX-809 (1 µM) glafenine and selected derivatives (compounds 49, 53, 54, 55, 56) (10 μM) and with BHK cells expressing wild-type CFTR (n = 4). (**E**) Relative intensity of bands B and band C in each lane in (**D**). Data in panels E is presented as the means ± SEM, n = 4.

To find a more potent version of glafenine, 55 analogs of glafenine were synthesized. (Suppl. Fig. 3 and Suppl. Methods). Glafenine derivatization focused on its dihydroxpropyl ester moiety. This moiety was chosen as it was the most amenable to derivatisation and that the two hydroxyls present here may have been susceptible to metabolism such as glucuronidation a known form of drug resistance^30,31^. Hence altering this site may slow the overly rapid metabolism of the glafenine compound and assist in its drug like characteristics. To determine whether the glafenine modifications were able to correct F508del-CFTR, we tested them using our HTS assay at 10 µM for 24 h (n = 4), (Fig. 2A). 14 were inactive for F508del-CFTR correction, and an additional 22 corrected F508del-CFTR but less than glafenine (10 µM). However, 20 compounds gave corrector signals that were larger than glafenine, with compounds 49 to 56 giving at least double that of glafenine (shown as red bars). Note that glafenine is compound 37 (green bar in Fig. 2A–C).

### Glafenine modifications improve the functional correction of F508del-CFTR

F508del-CFTR corrected by glafenine derivatives could yield partially misfolded, nonfunctional proteins and hence the level of surface CFTR may not be a true reflection of the amount of functional CFTR present. To determine if this was the case, we tested glafenine derivative-corrected CFTR in BHK cells using the FLIPR membrane potential (FMP) assay, which monitors membrane depolarization resulting from surface CFTR channel activation (Fig. 2B). We measured the F508del-CFTR response in cells incubated with each derivative at 10 µM for 24 h using 1 μM VX-809 as a control, (n = 4). Of the 20 derivatives with more correction than glafenine (Fig. 2A), 14 gave stronger CFTR functional responses to forskolin + genistein, indicating that the rescued mutant CFTR was functional with the strongest response provided by compound 49, which was 84% of the VX-809 signal.

We then monitored forskolin-stimulated short circuit current changes (*I*sc) in the polarized human-derived bronchial epithelial cell line CFBE41o − expressing F508del CFTR. Cells were treated with each compound (10 µM for 24 h) and measured in Ussing chambers during stimulation by forskolin + genistein (n = 4) (Fig. 2C). Among the 20 compounds with more corrector activity than glafenine (Fig. 2A), all 20 produced the same or greater levels of CFTR functional rescue. The strongest responses were obtained with compounds 49 to 56, in good agreement with the FMP assay (Fig. 2B). Responses to forskolin + genistein were abolished by CFTR_inh_-172, confirming that they were mediated by CFTR. Compound 49 provided the most functional F508del-CFTR correction at 35% (± 1.2%) VX-809, a nine-fold increase compared to glafenine.

Given the data, we chose to focus on the five most potent F508del-CFTR correcting derivatives (49, 53, 54, 55 and 56). Using the FMP assay in a concentration gradient (Suppl. Table 2) we determined the EC_50_ and the dose giving a maximal response (E_max_). The EC_50_ ranged between 1.12 μM and 40 nM, while E_max_ ranged between 9.6 and 1.4 μM (n = 4).

### Glafenine derivatives increase F508del-CFTR maturation

We next chose to study CFTR protein processing by utilizing immunoblotting (Fig. 2D) after BHK cells were treated with 10 µM for 24 h. Glafenine and derivatives that gave the strongest responses in Fig. 2A,B (49, 53, 54, 55 and 56) as well as VX-809 partially corrected F508del-CFTR processing (increased band C) (Fig. 2D, Suppl. Fig. 4). Derivatives 1 and 2 that did not show correction (Fig. 2A–C) were included as negative controls. The results of the immunoblot are completely consistent with the Ussing chamber with the compounds that produced the strongest signal in the Ussing chamber assays **(**Fig. 2C) also generated the most band C protein. While these results may not entirely match those in the surface expression assay what both assays show is the potency of the five selected glafenine derivatives. The difference between the amount of band C detected in Fig. 2D and the amount of surface CFTR found in Fig. 2A may be due to the possible trafficking of band B to the cell surface a phenomena that has previously reported elsewhere^32^. Quantification of the CFTR signal using ImageJ revealed that more than > 45% of F508del-CFTR was complex N-glycosylated (band C) following derivative treatment (Fig. 2E). Thus, glafenine and these five derivatives (49, 53, 54, 55 and 56) caused significant rescue of F508del-CFTR trafficking in BHK cells.

### The glafenine derivative functionally rescues F508del-CFTR in well-differentiated primary HBE cells

**Figure 3:**
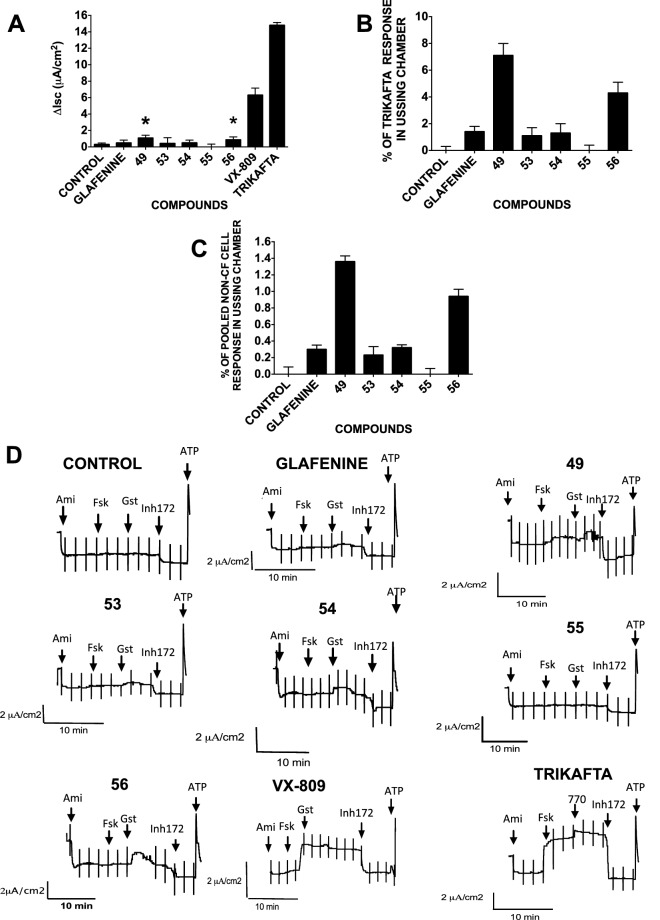
Demonstration of the ability of glafenine and selected derivatives to correct F508del-CFTR in primary human HBE cells. (**A**) F508del-CFTR functional expression in well-differentiated primary human bronchial epithelial (HBE) cells determined from the increase in short-circuit current. The basolateral membrane was permeabilized using nystatin, and an apical-to-basolateral chloride gradient was imposed. Representative I_sc_ responses of primary HBE cells expressing F508del-CFTR to sequential addition of 10 µM forskolin, 50 µM genistein, and 10 µM CFTRinh-172 after 24 h preincubation with 0.1% dimethylsulfoxide (vehicle), glafenine and compounds 49, 53, 54, 55 and 56 (10 µM), or VX-809 (1 μM) (n = 4). As a control Trikafta is tested with using VX = 770 (100 nM) instead of genistein. The asterisks mark glafenine derivatives (49, 56) that give a response significantly above the control level. (**B**,**C**) This is represented in two graphs, first as the change in current and second as the percentage of VX-809 response. (**D**) Additionally, included are the traces for each compound attained from the Ussing chamber in this experiment. Data in panels A,B and C are presented as the means ± SEM, n = 4, *p < 0.05, **p < 0.01 and ***p < 0.001.

Many correctors effective in non-polarized cells fail in well-differentiated polarized airway epithelial cells. To determine whether glafenine derivatives functionally correct F508del-CFTR in such cells (HBE), we tested glafenine and the five glafenine derivatives that gave the largest responses in CFBE41o- cells at 10 µM (Fig. 2C) (49, 53, 54, 55 and 56; Fig. 2C) and compared their response to those obtained using VX-809 at 1 µM in the Ussing chamber and Trikafta (n = 4) (Fig. 3). Compound 49 gave the most correction, 8% that of Trikafta (Fig. 3B), approximately 1.3% of non-CF HBE cells (Fig. 3C) and > fourfold higher than glafenine in well-differentiated HBE.

### Glafenine corrects CFTR by interacting with COX-2

**Figure 4:**
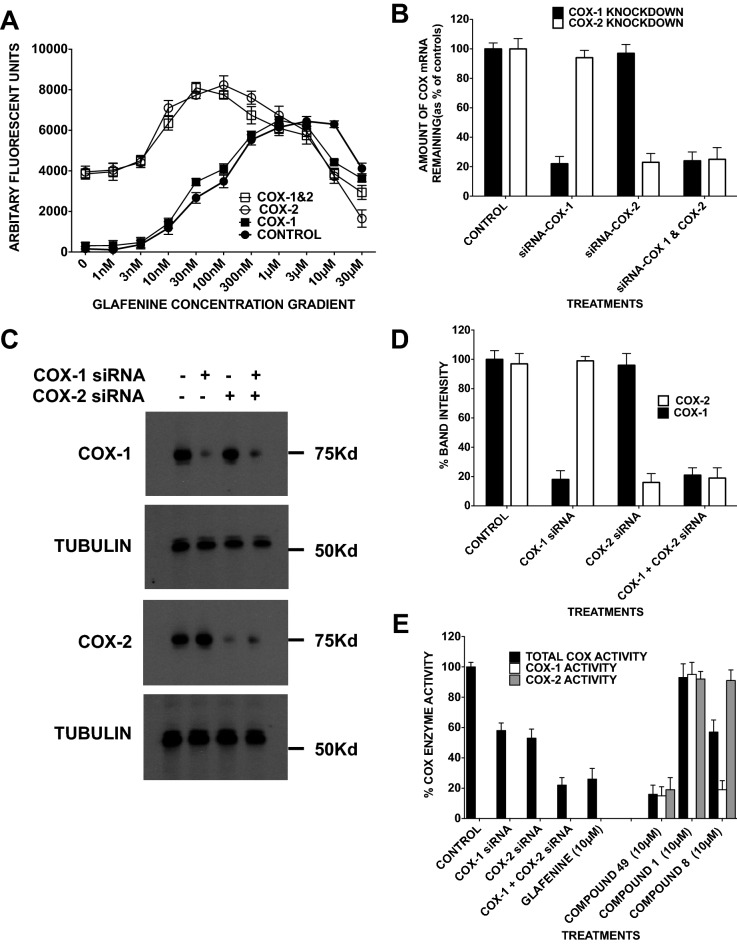
This study demonstrates that COX2 inhibition is required for glafenine-mediated CFTR correction and that glafenine interacts directly with COX2. (**A**) Cell surface CFTR in HEK cells expressing F508del-CFTR, treated with different concentrations of glafenine (0 nM, 1 nM, 3 nM, 10 nM, 30 nM, 100 nM, 300 nM, 1 µM, 3 µM, 10 µM, and 30 μM) in combination with siRNA knockdown, of control scrambled siRNA COX1 (filled square) COX2 (open circle), or both COX1 and COX2 (open square) for 24 h before 24-h incubation with glafenine (n = 4). (**B**) Q-PCR to show the effectiveness of siRNA to COX1 and COX2 both when used separately and together. (**C**) Immunoblots showing that the reduction in mRNA resulted in less COX1 and COX2 protein, as shown in (**B**). (**D**) Graph to show the relative intensity of bands B and band C in each lane in panel C as monitored by image J. (**E**) Graph to show the COX enzyme activity remaining in the cells 48 h after siRNA treatment for COX-1 and COX-2 separately and together. Also the COX enzyme activity detected upon 24 h treatment with glafenine and 3 analogs compounds 1, 8 and 49 all at 10 µM. Black bars represent total COX enzymatic activity measured from cell lysates, the white and shaded bars represent COX-1 and COX-2 enzymatic activity respectively obtained due to the inhibition of recombinant COX enzymes (n = 3). Data in (**B**), (**D**) and (**E**) are presented as the means ± SEM.

Broad-spectrum NSAIDs are known to inhibit both cyclooxygenase 1 (COX1) and cyclooxygenase 2 (COX2). To investigate whether a reduction in COX2 expression increases glafenine-mediated correction, we treated HEK cells expressing F508del-CFTR with siRNA against COX1 and COX2 separately and together for 24 h, treated them with varying glafenine concentrations (1 nM, 3 nM, 10 nM, 30 nM, 100 nM, 300 nM, 1 µM, 3 µM, 10 µM, and 30 µM) and monitored them for correction using the FMP assay (n = 4) (Fig. 4A–D, Suppl. Fig. 5). The optimal glafenine concentration for CFTR correction was 3 µM. When combined with siRNA targeting COX1 transcripts, optimal rescue was obtained using 1 µM glafenine. The glafenine concentration giving optimal F508del-CFTR correction after COX2 knockdown was 100 nM, which was significantly lower than that after COX1 knockdown. The role of COX2 was apparent even in the absence of glafenine, as the F508del-CFTR signal was enhanced in cells pretreated with siRNA against COX2. The fact that the maximal level of correction is increased upon the addition of glafenine may be explained by the fact that the siRNA to the COX enzymes does not totally remove the COX enzyme rather it reduces both the mRNA and the protein of COX 1 and COX 2 by approximately 80% (Fig. 4B–E). In particular as may be seen in Fig. 4E were COX enzymatic activity is measured. Given that both COX enzymes are expressed in the HEK cell line (Fig. 4C, Suppl. Fig. 5) then only the total COX activity can be measured here. Upon siRNA inhibition of COX enzyme expression for COX-1, the level of COX enzyme activity drops to 57% (± 5%) of the control COX activity and as may be seen in Fig. 4A there is no significant change in CFTR function. (See the 0 nM glafenine concentration point). In contrast when COX-2 siRNA is used although the level of COX enzyme activity drops to a similar level 55% (± 5%) of the control level Fig. 4A reveals CFTR function rises significantly (see the 0 nM glafenine concentration point) and a similar level of CFTR functional response is detected when both COX-1 and COX-2 are together reduced in expression by siRNA (Fig. 4A). Using siRNAs targeting both COX1 and COX2 further reduced the glafenine concentration needed for optimal F508del-CFTR correction from 100 to 30 nM. Hence, given that glafenine is an inhibitor of COX enzymes these results strongly supports the concept that glafenine rescue is mediated primarily through its reduction in COX2 function.

Further the COX enzyme activity was tested in the presence of three glafenine derivatives, (compounds 1, 8 and 49) both in HEK cell lysates and against recombinant COX enzymes (Fig. 4E) (n = 3). Compound 1 does not inhibit either COX-1 or COX-2 enzyme activity and as may be seen in Fig. 2A–C it does not rescue CFTR. In contrast compound 49 does inhibit both COX-1 and COX-2 and also corrects F508del-CFTR (Figs. 2, 3). Interestingly compound 8 does inhibit COX-1 but not COX-2 and like compound 1 does not correct F508del-CFTR (Fig. 2A–C). Indeed as may be seen by the black bars in Fig. 4E there is no significant differences between the total COX enzyme activity detected in the cells treated each either compound 8 or the siRNA to COX-1 and the activity in cell treated with COX-2 siRNA and yet only the COX-2 siRNA treated cells show significant F508del-CFTR correction. This evidence again suggests that there is a link between COX-2 inhibition in the arachidonic pathway and F508del-CFTR correction.

### Glafenine corrects F508del-CFTR via the arachidonic acid pathway

**Figure 5:**
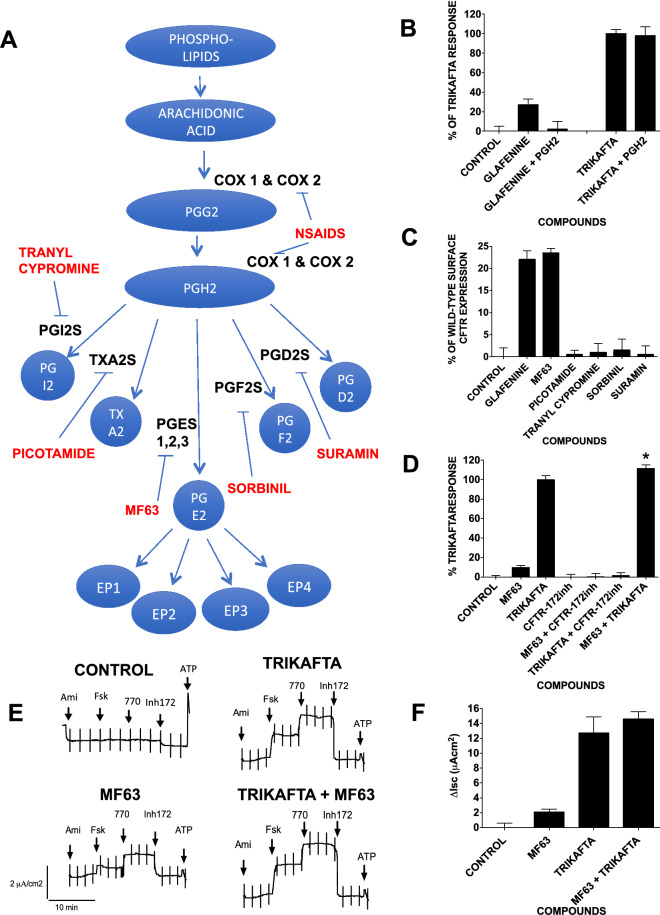
The mechanism of glafenine-mediated CFTR correction works via the arachidonic acid pathway. (**A**) A cartoon of the arachidonic acid pathway with specific enzyme inhibitors marked in red. (**B**) Cell-based HTS assay measuring surface F508del-CFTR in BHK cells after 24 h of treatment with glafenine, 10 μM and Trikafta in the presence and absence of PGH2 at 1 μM (n = 4). (**C**) Cell-based HTS assay measuring surface F508del-CFTR in BHK cells after 24 h of treatment with glafenine (10 μM), MF63 (10 μM), picotamide (1 μM), tranyl cypromine (10 μM), sorbinil (10 μM), and suramin (5 μM) (n = 4). (**D**) FMP assay (FMP) that monitors membrane depolarization induced by forskolin + VX-770 when cells are pretreated for 24 h with MF63 (10 μM) and Trikafta in the presence and absence of CFTR172inh (10 μM). Additionally, MF63 (10 μM) and Trikafta were added together for 24 h (n = 4). (**E**) Representative I_sc_ responses of primary HBE cells expressing F508del-CFTR functional expression in well-differentiated primary human bronchial epithelial (HBE) cells determined from the increase in short-circuit current stimulated by acute addition of forskolin + genistein (Δ*I*sc). The basolateral membrane was permeabilized using nystatin, and an apical-to-basolateral chloride gradient was imposed by sequential addition of 10 µM forskolin, 100 nM vx-770, and 10 µM CFTR inh-172 after 24 h of preincubation with 0.1% dimethyl sulfoxide (vehicle) and MF63 (10 µM). (**F**) Graph for each compound for the data attained from the Ussing chamber (**E**) (n = 4). Data in (**B**), (**C**), (**D**) and (**F**) are presented as the means ± SEM, n = 4, *p < 0.05, **p < 0.01 and ***p < 0.001.

Cyclooxygenase 2 is an enzyme in the phospholipid/arachidonic acid pathway (see Fig. 5A). To determine whether glafenine mediates CFTR correction via this pathway, we initially tested its ability to correct F508del-CFTR in the presence of excess prostaglandin H2 (PGH2). The experimental rationale is that if COX inhibition by glafenine reduces PGH2 production and promotes CFTR rescue, providing exogenous PGH2 should counteract this and reduce glafenine correction. To test this hypothesis, we treated BHK cells expressing F508del-CFTR with glafenine (10 µM) or Trikafta in the presence or absence of PGH2 (1 µM) for 24 h and then assessed F508del-CFTR rescue by measuring CFTR surface expression in our HTS assay (n = 4) (Fig. 5B). As shown in Fig. 5B, glafenine alone rescued CFTR, and this correction was abolished in the presence of PGH2; in contrast, PGH2 had no effect on the Trikafta mediated correction. These results suggest that a decrease in PGH2 is essential for glafenine-mediated F508del-CFTR correction.

The next step in the arachidonic acid pathway is PGH2 conversion into one of five effector molecules, with each reaction catalyzed by a different enzyme (see Fig. 5A). The five enzyme groups are prostaglandin E synthase 1,2,3 (PGES 1,2,3), thromboxane A2 synthase (TXA2S), prostaglandin I2 synthase (PGI2S), prostaglandin F2 synthase (PGF2S) and prostaglandin D2 synthase^33,34^. To establish which of these mediate F508del-CFTR rescue, we employed inhibitors specific for each of the five enzyme groups, i.e., MF63^35^, picotamide^36^, tranyl cypromine^37^, sorbinil^38^, and suramin^39^ were used at their published optimal dosages. The F508del-CFTR HTS surface expression assay was used to determine whether inhibiting any of these enzymes could recapitulate the rescue induced by knockdown or pharmacological inhibition of COX 2. While TXA2S, PGI2S, PGF2S and PGD2S had no effect on F508del-CFTR trafficking, inhibition of PGES 1, 2, and 3 by MF63 triggered F508del-CFTR trafficking to the same level as glafenine (n = 4) (Fig. 5C).

We also measured the effect of MF63 using the FMP functional assay both in the presence and absence of the CFTR-specific inhibitor CFTRinh172 (n = 4)^24^ (Fig. 5D). The MF63 produced F508del-CFTR correction that was modest (12.6% ± 1.9%) when compared to Trikafta, however the fact that this was abolished by the CFTR inhibitor, CFTRinh172, confirmed that it was CFTR channel function. Of note is that when used in combination, MF63 and Trikafta were additive (113.5% ± 2.3%), consistent with distinct mechanisms (n = 4) (Fig. 5D).

### F508del-CFTR corrected in primary HBE cells by MF63 is functional

To determine whether MF63 and hence inhibition of PGES 1, 2, and 3 functionally correct F508del-CFTR in human primary bronchial epithelial cells (HBE), we also monitored stimulated short circuit current (*I*_SC_) in Ussing chambers (n = 4) (Fig. 5E,F). The results show that the functional rescue of F508del-CFTR by MF63 is very similar to that provided by glafenine and to that seen in the FMP assay earlier Fig. 5D). However, unlike Fig. 5D although the combination of MF63 and Trikafta increases the amount of F508del-CFTR correction it is not significant. These results indicate that glafenine and its analogs correct F508del-CFTR by inhibiting the conversion of arachidonic acid to PGH2 through its intermediate PGG2, which leads to diminished formation of PGE2 from PGH2 by the enzyme PGES 1,2,3.

### F508del-CFTR correction by MF63 works by EP4

**Figure 6:**
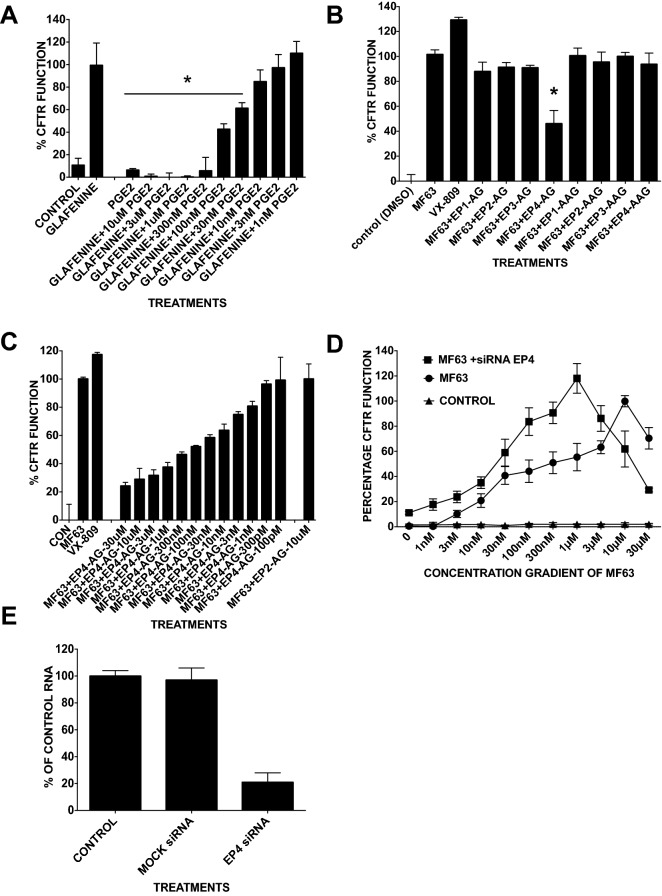
The mechanism of glafenine-mediated CFTR correction works via the inhibition of prostaglandin E_2_ receptor 4 (EP4) activation. (**A**) FMP assay that monitors membrane depolarization induced by forskolin + genistein when BHK cells are pretreated with glafenine (10 µM) and various concentrations of the arachidonic pathway derivative prostaglandin E2 for 24 h prior to assaying (n = 4). (**B**) FMP assay (FMP) of BHK cells pretreated for 24 h with MF63 (10 µM) and an agonist (AG) and antagonist (AAG) for each of the four EP receptors (agonists for EPs 1 to 4 are ONO-D1-OO4, butaprost, sulprostone and CAY10598, respectively; for the antagonists, they are SC-51089, PF0044189948, L798106 and ONO-AE3-208, respectively) (10 μM) performed in BHK cells expressing F508del-CFTR (n = 4). (**C**) FMP assay in cells pretreated for 24 h with MF63 (10 µM) and a range of concentrations (10 µM to 100 pM) of the EP4 agonist CAY10598. The vertex corrector VX-809 (3 µM) was used as a positive control, and the EP2 agonist butaprost (10 µM) was used in combination with MF63 to show that the effects seen with the EP4 agonist were specific to EP4 (n = 4). (**D**) FMP assay in HEK cells expressing F508del-CFTR, treated with different concentrations of MF63 (0 nM, 1 nM, 3 nM, 10 nM, 30 nM, 100 nM, 300 nM, 1 µM, 3 µM, 10 µM, and 30 μM) alone (filled circle) or in combination with siRNA knockdown, of control scrambled siRNA (filled rhombus) or siRNA to EP4 (filled square) for 24 h before 24-h incubation with MF63 (n = 4). (**E**) Q-PCR to show the effectiveness of siRNA to EP4.

The results raise the question of whether glafenine/MF63-mediated CFTR correction proceeds via the restriction of PGE2 production and whether the addition of PGE2 disrupts this CFTR rescue. This was tested in BHK cells expressing F508del-CFTR by adding various amounts of PGE2 (10 µM to 1 nM) at the same time as glafenine (10 µM) incubation for 24 h and testing CFTR functionality by FMP assay (Fig. 6A). The results demonstrated significant inhibition of glafenine mediated CFTR correction at PGE2 concentrations down to 30 nM. Given the similarities in effect generated by PGE2 and PGD2 a similar experiment was undertaken to test the effect of PGE2 and PGD2 on both Glafenine and Trikafta-mediated CFTR correction. No significant disruption of CFTR correction was seen for Trikafta and glafenine correction was only disrupted by PGE2 (n = 3) (Suppl. Figs. 6 and 7). These results further support the idea that glafenine/MF63-based CFTR correction occurs via the reduction of PGE2 present in the cell.

The next stage in the arachidonic pathway is the stimulation of one of the 4 prostaglandin E2 receptors (EP1, EP2, EP3 and EP4) by PGE2. To determine whether CFTR correction by MF63 proceeds along this pathway, specific agonists and antagonists were employed. Specifically, the agonist for EP1 was ONO-D1-004^40^ for EP2 Butaprost^41^ for EP3 Sulprostone^42^ and for EP4 CAY10598^43^ and the antagonists for EP1 SC-51089^44^ for EP2 PF004418948^45^ for EP3 L798106^46^ and for EP4 ONO-AE3-208^47^. BHK cells were treated with MF63 in the presence of each of these agonists and antagonists separately. Only the agonist for EP4 (CAY10598) reduced the effectiveness of MF63 as a corrector of F508del-CFTR with a reduction to 22% of MF63 alone (Fig. 6B,C), suggesting that the downstream activation of the arachidonic pathway only via EP4 negated the ability of MF63 to rescue F508del-CFTR. To confirm this finding, this experiment was repeated over a range of EP4 agonist concentrations of 10 μM to 100 pM (n = 4) (Fig. 6C). The results demonstrated a level of dose dependency, with the more agonists there was, the greater the reduction in MF63 correction although this does appear to begin to plateau at around 3 μM. To further investigate the role of EP4 in MF63-mediated correction, we treated HEK cells expressing F508del-CFTR with siRNA against EP4 for 24 h, treated them with varying MF63 concentrations (1 nM, 3 nM, 10 nM, 30 nM, 100 nM, 300 nM, 1 µM, 3 µM, 10 µM, and 30 µM) and monitored them for correction using the FMP assay (n = 4) (Fig. 6D). The optimal MF63 CFTR correction concentration was 10 µM. When combined with siRNA targeting EP4 transcripts, optimal inhibition was obtained using 1 µM MF63. F508del-CFTR function under basal conditions (i.e., in the absence of MF63) was elevated whenever EP4 expression was reduced, again suggesting a link between EP4 activity and CFTR trafficking. Such changes in the MF63 optimal dose were achieved by reducing the amount of EP4 mRNA by 82%, as measured by Q-PCR (Fig. 6E). Together, these results strongly suggest that glafenine-mediated CFTR correction works specifically via the inhibition of EP4.

### Glafenine and its derivatives rescue other class 2 CFTR mutations

Class 2 mutations cause retention of misfolded CFTR in the ER, leading to premature degradation of the protein. VX-809 (Lumacaftor) and its structural analog VX-661 (tezacaftor) are both correctors that interact with the mutated NBD-1 domain of F508del-CFTR^48–51^. Orkambi, the first CFTR modulator therapy to include a corrector, is a combination of the corrector Lumacaftor and the CFTR potentiator VX-770 (Ivacaftor). Some class 2 mutations, such as G85E (the 4^th^ most common CF mutant) or N1303K (15^th^ most common CF mutant^21^), are not corrected by VX-809^19,20,22^.

**Figure 7:**
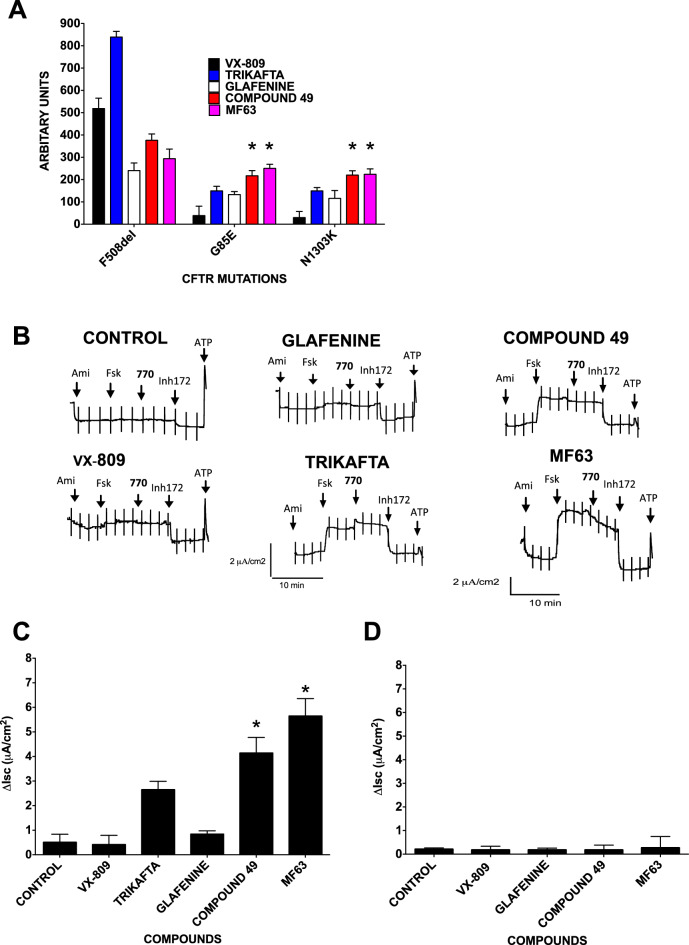
Primary HBE cells revealed that glafenine, compound 49 and MF63 are potent correctors of class 2 CFTR mutations. **(A**) FMP assay (FMP) that monitors membrane depolarization induced by forskolin + genistein when cells are pretreated for 24 h with glafenine, its derivative, compound 49 and MF63 (all at 10 μM) and separately with VX-809 (1 µM) and Trikafta, performed in Fischer rat thyroid (FRT) expressing a selection of class 2 CFTR mutations (F508del-, G85E, and N1303K), (n = 3). (**B**) G85E-CFTR functional expression in well-differentiated primary human bronchial epithelial (HBE) cells determined from the increase in short-circuit current stimulated by acute addition of forskolin + VX-770 (Δ*I*_sc_), (NB It should be noted that the cells of this patient were heterozygotic in which one allele expressed G85E-CFTR and the other expressed the class 1 type mutation 621-1GT-CFTR). Representative I_sc_ responses of primary HBE cells expressing G85E-CFTR to sequential addition of 10 µM forskolin, 100 nM VX-770, and 10 µM CFTRinh-172 after 24 h preincubation with 0.1% dimethyl sulfoxide (vehicle), glafenine (10 µM) or VX-809 (1 μM), compound 49 (10 μM) and MF63 (10 μM), and Trikafta (N = 3). (**C**) Graphical representation of the Ussing chamber experiment outlined in (**B**). (**D**) Class 1 type 621-1GT-CFTR functional expression in well-differentiated primary human bronchial epithelial (HBE) cells determined from the increase in short-circuit current stimulated by acute addition of forskolin + genistein (Δ*I*sc). Representative I_sc_ responses of primary HBE cells expressing 621-1GT-CFTR to sequential addition of 10 µM forskolin, 50 µM genistein, and 10 µM CFTRinh-172 after 24 h preincubation with 0.1% dimethylsulfoxide (vehicle), VX-809 (1 μM), glafenine, compound 49, and MF63 (all at 10 µM), (n = 3). Data in A and D are presented as the means ± SEM, n = 4, *p < 0.05, **p < 0.01 and ***p < 0.001.

We tested whether these rare mutations respond to glafenine since unlike pharmacological chaperones, they do not require direct interaction with CFTR to correct. Fischer rat thyroid (FRT) cells expressing these mutants were pretreated with glafenine, compound 49 VX-809 or Trikafta^21^. All these compounds partially corrected the trafficking of these class 2 mutations and restored some anion conductance according to the FMP assay. Moreover, glafenine and its analogs rescued more CFTR than either VX-809 or Trikafta for mutants other than F508del-CFTR (n = 5) (Fig. 7A), and compound 49 was more effective than glafenine. Indeed, for both G85E and N1303K the response obtained by glafenine and compound 49 was significantly higher than the correction attained by either VX-809 or Trikafta.

### G85E-CFTR is rescued by glafenine in primary HBE cells

To determine whether glafenine can correct class 2 mutants that do not respond to VX-809 in HBEs, we measured *Iscs* across well-differentiated primary HBE cells in Ussing chambers (n = 3) (Fig. 7B, Suppl. Fig. 8). Although the G85E mutation is rare, we were able to study it by using HBE cells from a patient heterozygous for G85E and a class 1 mutation (621 + 1GT) that does not produce full-length CFTR as the other allele. The results were striking, as while VX-809 (3 µM) gave no response, and Trikafta did give a response both compounds 49 and MF63 gave robust correction at 10 μM that was significantly greater then Trikafta (9.5% and 13.4% of WT, respectively as opposed to 6.1% for Trikafta, (n = 3) Fig. 7B,D). Glafenine also gave some correction but with a modest response (0.8% of WT). As a negative control, we also tested these correctors using HBE cells from a patient homozygous for the 621-1GT mutation (Fig. 7C). As expected none of the compounds increased *Isc*. As in this form of CFTR, alternative splicing occurs (with the use of a cryptic donor sequence TT_528_/GTGAGG) in exon 4. Hence, even though the open reading frame is preserved, large sections of the first and second transmembrane and subsequent cytoplasmic domains are missing from this CFTR protein^52^. Therefore, even if 621-1GT-CFTR does reach the plasma membrane, it displays no channel activity. Thus, when functional CFTR activity is detected in HBE cells expressing G85E and 621-1GT CFTR, this functionality is derived solely from the G85E allele.

In conclusion, glafenine analogs that inhibit the arachidonic pathway upstream of the prostaglandin E synthase 2 enzyme (PGES2) are a novel mechanism for the correction of G85E CFTR in HBE cells.

## Discussion

CFTR class 2 mutations produce misfolded proteins that are recognized by cellular quality control systems, retained in the ER, retrotranslocated into the cytoplasm, ubiquitinated and degraded by the proteasome^3^. Effective CFTR potentiators and correctors for F508del-CFTR now exist. The triple combination drug Trikafta (Tezacaftor + Elexcaftor + Ivacaftor) provides significant clinical benefit to patients with F508del-CFTR (e.g., an increase in lung function of ~ 13.8% as measured by FEV1). Some reports suggest that Trikafta can rescue rarer class mutants such as N1303K and G85E. However, recent research has found that while trikafta can rescue some rare class 2 mutations, such as M1101K, G85E and N1303K, the level of rescue for G85E and N1303K is modest and possibly not clinically beneficial^14^. Indeed, if the previous widely used CFTR modulator combination Orkambi is any guide, then a significant percentage of class 2 CF patients will remain unresponsive or not benefit from the treatment^18–20^. Such results have led to theratyping the concept where some CFTR mutations respond to drugs better than others in the same class. This in turn has triggered a reorganization of the CFTR mutants based on their responses to corrector/potentiator compounds^53^, and it has inspired the search for more pharmacological chaperone correctors targeted to new CFTR binding sites to test them in combinations that rescue previously recalcitrant CFTR mutants such as G85E^15,29^. This raises the prospect of requiring dozens of pharmacological chaperones to rescue all class 2 CFTR mutations. To avoid such a situation, we turned to proteostasis modulators in search of a mutation-insensitive corrector that could rescue multiple CFTR class 2 mutations.

Diverse proteostasis modulators that increase F508del-CFTR trafficking have been identified in cell-based screens^10,54^. Although some phosphodiesterase inhibitors have well-known targets, in most instances, the link to protein quality control is obscure^27^. We have reported previously on the ability of one NSAID, ibuprofen, to act as an F508del-CFTR corrector^26^.

In this study, we examined several NSAID classes for their abilities to rescue F508del-CFTR (Suppl. Table 1). We found that 7 out of the 8 groups tested had members that could correct F508del-CFTR, and of these, glafenine was one of the most potent. Interestingly, one group of NSAIDs that did not correct F508del-CFTR was salicylates. This may be due to the previously reported inhibition of CFTR expression by members of this group^55^.

We tested the ability of the NSAID glafenine to correct F508del-CFTR (Fig. 1D,E) and showed that F508del-CFTR can traffic from the ER to the Golgi, becoming the band C form, which is the N-glycosylated form found at the plasma membrane. In this experiment, 26% of F508del-CFTR was detected as band C and was approximately 30% of that obtained for wild-type CFTR (Fig. 1E). Furthermore, glafenine showed little inhibition of CFTR expression. The results in Fig. 1 also demonstrated that glafenine, unlike VX-809 or RDR1^29^, is a proteostasis modulator and can act additively with pharmacological chaperones such as VX-809 and those found in Trikafta to improve the correction of F508del-CFTR.

We increased the potency of glafenine by synthesizing 55 derivatives of glafenine and testing them as F508del-CFTR trafficking correctors. Depending on the assay, between 20 and 30 of the derivatives gave a more potent F508del-CFTR corrector response than glafenine. Compound 49 gave the most efficacious response, with 36% VX-809, fourfold more than glafenine. These are encouraging results and promising for a future SAR program. Glafenine was withdrawn from widespread clinical use due to reports of hepatotoxicity^28^. This represents another reason for future SAR to develop a glafenine analog that does not display these issues.

It should be noted that in this work we utilized both genistein and separately VX-770 as potentiators. In the case of genistein this may conceivably be a concern as genistein has been reported as a tyrosine kinase inhibitor^56^ which may disrupt the arachidonic pathway and hence alter the cellular response regardless of the other treatment. However, we saw no evidence for this in our controls for those experiments. Further when we compare the glafenine responses we see in genistein-potentiated cells to those potentiated with VX-770 we see no difference in response or trend relative both the positive and negative controls. Hence this suggests that genistein has no effect on the arachidonic pathway that would alter NSAID mediated CFTR correction.

Glafenine is not the only NSAID reported to correct F508del-CFTR, and ibuprofen, sulindac and fulfinindac have also been reported to correct^26,57^. Our findings with siRNAs targeting COX1 and COX2 (Fig. 4) support the idea that the interaction with COX2 is key to CFTR rescue. The idea that it is COX-2 rather than COX-1 is supported by the finding that compound 8 which strongly inhibits COX-1 but not COX-2 does not correct F508del-CFTR. Further, the COX-2 specific inhibitor celecoxib rescues the mislocalization of F508del-CFTR but the COX-1 specific inhibitors indomethacin and prioxicam do not rescue CFTR (Sup. Table 1). Indeed only COX2 knockdown reduced the glafenine concentration needed for optimal correction (3 µM to 100 nM) (Fig. 4A). This raises the possibility that COX2 inhibition helping to correct F508del-CFTR may be part of a feedback loop given that CFTR expression suppresses COX 2 expression^58^.

NSAID mediated trafficking correction occurs through disruption of aspects of the arachidonic acid pathway in particular by the inhibition of COX2. Circumventing COX2 inhibition by supplying exogenous prostaglandin H2 abolished glafenine correction but had no effect on CFTR rescue by Trikafta, confirming that PGH2 reduction underlies correction by glafenine. Furthermore, studies have revealed that the conversion of PGH2 to PGE2 by PGE2S is the key to CFTR correction and that MF63, a PGE2S-specific inhibitor, is also a CFTR corrector (Fig. 5D). Ultimately, the glafenine-derived F508del-CFTR correction pathway occurs through the reduction in prostaglandin E2 receptor 4 (EP4) stimulation and not other EP receptors (Fig. 6).

The dysregulation of fatty acids and the arachidonic acid pathway has been reported in cystic fibrosis^59^. Indeed, arachidonic acid accumulation may inhibit CFTR potentially by binding CFTR on the cytoplasmic side and interacting with the positively charged residues K95 and R303^60^. This finding may explain the improved G85E-CFTR correction by MF63 in HBE cells when compared with compound 49 (13.4% of non-CF response for MF63 and 9.5% for compound 49). MF63 is a specific inhibitor of PGE2S enzymes and not COX1 or 2. Hence, it does not entirely block the arachidonic pathway from arachidonic acid metabolism via the TXA2S, PGI2S, PGF2S and PGD2S enzymes. Arachidonic acid levels are reduced with MF63, so there may be less CFTR inhibition^61^. This is important, as arachidonic acid levels are known to be elevated in CF patients^60,62^. Furthermore, the continued presence of the other branches of the arachidonic pathway in particular PGD2 may also offset PGE2 loss in terms of the cAMP activation of cell surface CFTR, which has been reported in mice^63^.

The goal here is to develop therapies for pleiotropic CFTR mutations. We discovered that the glafenine derivative compound 49 can partially correct at least 2 other CFTR mutants (G85E and N1303K) in addition to F508del and is more effective than either VX-809 or Trikafta (Fig. 7A). To establish the capabilities of glafenine, compound 49 and MF63 as correctors of class 2 mutations, we tested the correction of G85E-CFTR in human primary HBE cells (Fig. 7B,D and Suppl. Fig. 3). We used heterozygous HBE cells in which one allele expressed the class 1 mutation 621-1GT and the other expressed the G85E mutation (Fig. 7C). Glafenine corrected this mutant protein better than VX-809, but the level was modest. However, both compound 49 and MF63 together gave significant G85E-CFTR correction (9.5% and 13.4% of non-CF patient cells). The correction detected in these heterozygous HBE cells was due entirely to the rescue of the single G85E allele, as the 621-1GT-CFTR allele produces a truncated CFTR with no ion channel^52^. This correction of the G85E mutation joins other allele-specific correctors, such as Ivacaftor for G551D and Lumacaftor (and Trikafta) for F508del, both of which are considered paradigms for personalized medicine.

The results are promising, as glafenine and its derivative compound 49 are early developmental compounds with preliminary medicinal chemistry and one in which a clear structure–activity relationship has yet to be determined. The use of mutation neutral proteostasis correctors of CFTR mutations such as glafenine and MF63, together with pharmacological chaperones such as VX-809 and Trikafta, may hold considerable promise as adjunct therapies for hard-to-treat CFTR mutations.

The development of proteostasis modulators that target metabolic pathways rather than bind a particular mutant raises the possibility of commonalities in how disparate proteins can be rescued. Interestingly, reports suggest that glafenine may be effective in folding defects in SLC4A11, a protein that, when mislocalized, causes corneal dystrophy^64^. An NSAID, perhaps such as glafenine, could be the basis for a treatment for multiple protein-misfolding and trafficking diseases.

## Materials and methods

### Reagents

Compounds were purchased from commercial sources as follows: VX-809, VX-661 and VX-445 (S1565, S7059 and S8851 all from Selleckchem Houston, Texas, USA), RDR1 (STK001879 Vitas-M laboratory Champaign, IL. USA), glafenine (G6895 Sigma-Aldrich St. Louis, MO USA), synthesis and structural confirmation of glafenine derivatives were performed at GlaxoSmithKline, Stevenage, UK (see Supplementary Methods section and Supplementary Fig. S1). Prostaglandin E_2_ receptor agonists were from as follows: ONO-D1-004 (100 nM) for EP1, Butaprost (10 µM) for EP2, Sulprostone (100 nM) for EP3 (all Sigma) and Cay10598 (10 nM) (Cayman chemicals) for EP4 and the antagonists were from sc-10589 (100 nM) for EP1 (Cayman Chemicals) PF-004418948 (100 nM) for EP2, L798106 (10 nM) for EP3 and ONO-AE3-208 (10 nM) for EP4 (all Sigma). The amounts stated in the brackets beside each compound are the final concentrations used in the experiments and correspond to the optimal dosages used in the literature apart from this unless otherwise stated. All compounds were used at 10 µM except VX-809, which was used at 1 µM. The amount of free VX-770 varies greatly depending on the presence of albumin in the medium^65^. If no albumin is present in the medium as is the case in our using chamber experiments, then the Trikafta composition used was composed of 3 µM VX-661, 3 µM VX-445 and 10 nM VX-770 (if cells were being treated for 24 h) for acute short-term treatments 100 nM VX-770 was used. If however, albumin is present in the medium then Trikafta is composed of; 2 µM VX-445, 18 µM VX-661 and 1 µM VX-770. Prostaglandin H2 and prostaglandins E2 and D2 were all purchased from Sigma.

### Cells

Class 2 CFTR mutant cDNAs (F508del-, G85E, and N1303K) were stably expressed in the FRT (Fischer Rat Thyroid) using the Flip-in system (Thermo Fisher) and were provided by Drs. Jeong Hong and Eric Sorscher, Emory University^53^. CF primary HBE cells were obtained from the Primary Airway Cell Biobank at McGill University and were isolated from tissues obtained from the Centre Hospitalier de l’Université de Montréal with support from Cystic Fibrosis Canada and the Respiratory Health Network of the Fonds de Recherche du Québec-Santé. The tissues were obtained at transplantation after informed written consent following protocols approved by the Institutional Review Boards of the CHUM and McGill University. All methods utilizing primary HBE cells were performed in accordance with relevant guidelines and regulations of the Institutional Review Boards of the CHUM and McGill University (IRB review number A08-M70-14B).

### High throughput screening assay

The high-throughput screening (HTS) assay was performed as previously reported^10,24^. Briefly, F508del-CFTR bearing three tandem hemagglutinin epitopes (3HA) in the fourth extracellular loop was stably expressed in baby hamster kidney (BHK) cells, plated in 96-well plates, and treated with test compounds for 24 h. Cells were then fixed and immunostained using a mouse monoclonal anti-HA antibody (Sigma-Aldrich, St. Louis, MO) for quantification of surface CFTR. Hits were those compounds that lacked intrinsic fluorescence and that gave signals that were consistently three standard deviations higher than untreated control cells.

### Antibodies

The anti-CFTR antibodies used were sc-10747, anti-CFTR (Santa Cruz Biotechnology, Dallas TX), and anti-CFTR MAB3484 (Millipore, Etobicoke, ON Canada). The COX1 antibody used was the anti-COX1 antibody EPR5866 (AB109025 Abcam Cambridge, UK), and the COX-2 antibody used was the anti-COX-2 antibody EPR12012 (AB179800 Abcam Cambridge, UK) and hemagglutinin tag (H-9658 Sigma St. Louis, MO) was used.

### Immunoblot

Total protein was quantified in cell lysates using the Bradford assay (BioRad), separated by SDS-PAGE (6% polyacrylamide gels), and analyzed by Western blotting as described previously. Western blots were blocked with 5% skimmed milk in PBS and probed overnight at 4 °C using the monoclonal primary anti-CFTR antibody (see “Antibodies” section above) diluted 1:1000. Blots were washed four times in PBS before adding the secondary HRP-conjugated anti-mouse antibody at a dilution of 1:15,000 (Amersham) for 1 h at room temperature, washed again five times in PBS and visualized using chemiluminescence (Pierce). The relative intensity of each CFTR glycoform (band B or band C) was measured by densitometry using ImageJ software^66^ and reported as the percentage of total CFTR normalized to the amount of tubulin in the same lane (i.e., B + C) and normalized to the amount of tubulin.

### Primary human bronchial epithelial (HBE) cells

Primary human bronchial epithelial (pHBE) cells were obtained by the Primary Airway Cell Biobank in the Cystic Fibrosis Translational Research Centre at McGill University using CF lung tissue provided by the Centre Hospitalier de l’Université de Montréal biobank of respiratory tissues after written informed consent was given by the donors. All procedures were approved by the Institutional Review Board of McGill University (A08-M70-14B). Cells were isolated following methods described by Fulcher et al.^67^ (cultured in T75 flasks in Bronchial Epithelial Cell Growth Medium (Lonza), and incubated at 37 °C in 5% CO_2_-95% O_2_. For regular experiments, cells were seeded on collagen IV-coated permeable Transwell supports (Corning) and cultured in air–liquid interface (ALI) medium^67^. After 3 days, the apical medium was removed, and cells were allowed to differentiate for 28 days at the air–liquid interface before study.

### Voltage-clamp studies of primary human bronchial epithelial (HBE) cells

HBE cells were seeded onto fibronectin-coated Snapwell inserts (Corning, Tewksbury MA), and the apical medium was removed after 24 h to establish an air–liquid interface^68^. Transepithelial resistance was monitored using an EVOM epithelial volt-ohmmeter (World Precision Instr. Sarasota FL), and monolayers were used after 4 weeks when the resistance was 300–400 Ω cm^2^. HBE monolayers expressing F508del-CFTR were treated on both sides with 0.1% dimethylsulfoxide (negative control) or 10 μM test compound (except VX-809; 1 μM) in OptiMEM containing 2% (v/v) fetal bovine serum. The short-circuit current (*I*sc) was measured across monolayers mounted in modified Ussing chambers and was voltage clamped using a VCCMC6 multichannel current–voltage clamp (Physiologic Instruments San Diego CA.).

Apical membrane conductance was functionally isolated by permeabilizing the basolateral membrane with 200 μg/ml nystatin and imposing an apical-to-basolateral Cl^−^ gradient. The basolateral solution contained 1.2 mM NaCl, 115 mM Na-gluconate, 25 mM NaHCO3, 1.2 mM MgCl_2_, 4 mM CaCl_2_, 2.4 mM KH_2_PO_4_, 1.24 mM K_2_HPO_4_, and 10 mM glucose (pH 7.4). The apical solution contained 115 mM NaCl, 25 mM NaHCO_3_, 1.2 mM MgCl_2_, 1.2 mM CaCl_2_, 2.4 mM KH_2_PO_4_, 1.24 mM K_2_HPO_4_, and 10 mM mannitol (pH 7.4). Apical glucose was replaced with mannitol to eliminate current mediated due to Na^+^-glucose cotransport. Successful permeabilization of the basolateral membrane under these conditions was obvious from the reversal of *I*sc. Solutions were maintained at 37 °C and continuously stirred by gassing with 95% O2/5% CO2. The transepithelial voltage was measured, and currents passed through agar bridge Ag/AgCl electrodes. Pulses (1 mV amplitude, 1 s duration) were delivered every 90 s to monitor resistance, and a PowerLab/8SP interface was used for data acquisition. CFTR was activated by adding 10 µM forskolin + 50 µM genistein to the apical bathing solution, and the resulting Isc was sensitive to CFTR inh-172 [10 µM]^69^, confirming that it was mediated by CFTR.

### FLIPR membrane potential assay (FMP assay)

A voltage-sensitive assay was used to assay the functional correction of F508del-CFTR in BHK and FRT cells. Cells were preloaded with a derivative of the voltage-sensitive FLIPR fluorescent dye bis-(1,3-dibutylbarbituric acid) trimethine oxonol [DiBac_4_]) (https://www.google.com/patents/US6420183), which enters the plasma membrane of the cell and is quenched by the addition of a proprietary quencher to the medium. Activation of CFTR depolarizes the plasma membrane, and the dye moves to the inner leaflet of the membrane, which relieves quenching and increases fluorescence. Assays were performed in 96-well plates containing 25,000 cells per well in 100 μl of medium. Cells were incubated with test compound for 24 h, washed with PBS, exposed to 70 μl dye solution that also contained genistein (GST; 50 µM), and incubated at room temperature for 5 min. Dye solution was prepared in low chloride buffer containing 160 mM Na gluconate, 4.5 mM KCl, 2 CaCl_2_, 1 mM MgCl_2_, 10 mM D-glucose and 10 mM HEPES, pH 7.4. After adding GST and mixing gently with a pipette, the 96-well plate was placed in a plate reader to measure fluorescence (emission 565 nM). Forskolin (10 µM) was then added, and the increase in signal was used to calculate the mean initial rate (i.e., Vmax) as a measure of CFTR function^70^. The mean initial rate for each treatment with appropriate units was determined.

### Cellular thermal stability assay

BHK cells expressing F508del-CFTR were cultured until they reached 90% confluence. They were then harvested and washed twice in PBS, incubated on ice for 10 min, and centrifuged at 1200×*g* at 4 °C for 10 min to pellet the cells. The cell pellet was then resuspended in lysis buffer (PBS containing 0.4% v/v Triton X-100 and Roche protease inhibitor cocktail) and incubated for 20 min on ice with occasional vortexing. The lysate was then transferred into separate PCR tubes in 40 μl aliquots, and the test compound was added at a final concentration of 1 or 10 µM depending on the compound and incubated for 5 min on ice. Tubes were then placed in a thermocycler and heated to a range of temperatures (33, 38, 43, 47, 52, 57 and 61 °C) for 10 min. The lysate was collected into a fresh tube and centrifuged for 10 min at 10,000×*g* at 4 °C. The supernatant and pellet were both collected, and the pellet was immunoblotted. The thermal shift of protein stability is determined by the temperature at which the protein of interest (F508del-CFTR or COX2) appeared in the pellet fraction^71–73^.

### High-throughput siRNA assay

For siRNA knockdown, human embryonic kidney 293 (HEK293) cells expressing F508del-CFTR-3HA or wild-type CFTR-3HA were used. COX-1- and COX-2-specific or nontargeting siRNAs were arrayed into a 96-well plate and used for transduction as described in the manufacturer’s instructions. HEK CFTR wild-type cells were added to the plate as controls. The next day, the medium was exchanged for fresh medium containing antibiotics and glafenine (at concentrations between 1 nM and 30 µM) or dimethylsulfoxide. Surface expression was analyzed 24 h later, as described elsewhere^27^**.** A similar approach was undertaken for the siRNA knockdown of EP4 except that MF63 was used instead of glafenine, and CFTR function was assayed using the FMP assay outlined above.

### COX enzyme assays

COX enzymatic assays were performed on lysates from human embryonic kidney (HEK293) cells expressing F508del-CFTR-3HA cells pretreated with siRNA for COX-1 and/or COX-2 for 48 h or on glafenine analog compounds using recombinant COX-1 and/or COX-2. The assays used were the commercial cyclooxygenase 1 (COX1) inhibitor screening assay kit (Abcam 204698) and separately in a COX-2 inhibitor Screening Kit (Millipore/Sigma MAK399) as per the manufacturers instructions (both assay kit provided recombinant COX enzyme for the assay). Both assays are based on a 96 well plate format on the principle of measuring the amount of prostaglandin G2 an intermediate product generated by COX enzyme activity fluorescently at excitation 535 nm at emission 587 nm. With regard to the cellular lysates only a total COX enzymatic activity could be determined as both COX-1 and COX-2 were present in the lysate (see Fig. 4C).

### Statistics

All results are expressed as the mean ± SEM of n observations. Data sets were compared by analysis of variance (ANOVA) or Student’s t-test using GraphPad Prism version 4. Differences were considered statistically significant when p < 0.05. ns: not-significant difference, *p  < 0.05, **p < 0.01, ***p < 0.001.

## Supplementary Information

Supplementary Information.
