# Supplementary material for: The NSAID glafenine rescues class 2 CFTR mutants via cyclooxygenase 2 inhibition of the arachidonic acid pathway
Source: Sci Rep. 2022 Mar 17;12:4595. doi: 10.1038/s41598-022-08661-8 (PMC8930988; doi:10.1038/s41598-022-08661-8)
Supplement: Supplementary file 1 — Supplementary Information. [file 41598_2022_8661_MOESM1_ESM.docx]

**Supplementary Materials for**

**The NSAID Glafenine rescues class 2 CFTR mutants via Cyclooxygenase 2 inhibition of the arachidonic acid pathway.**

Graeme W. Carlile^1*^, Qi Yang^1^, Elizabeth Matthes^2^, Jie Laio^2^, Véronique Birault ^3^, Helen F. Sneddon ^4^, Darren L. Poole ^5^, Callum J. Hall ^5^ , John W. Hanrahan^2^ and David Y. Thomas^1^

^1^Cystic Fibrosis Translational Research Centre, Department of Biochemistry, Department of Human Genetics, McGill University, Montréal, Québec Canada, H3G 1Y6.

^2^ McGill Cystic Fibrosis Translational Research Centre, Department of Physiology McGill University Montreal Québec Canada, H3G 1Y6.

^3^Translation Dept. The Francis Crick Institute, 1 Midland Road, London, United Kingdom, NW1 1AT.

^4^Green Chemistry Centre of Excellence, Dept. of Chemistry, University of York, Heslington, York. United Kingdom, YO10 5DD,

^5^Medicinal Chemistry GlaxoSmithKline, Gunnels Wood Road, Stevenage, Herts., United Kingdom, SG1 2NY.

- Corresponding Author; Graeme W. Carlile

**The PDF File includes:**

Figs. S1 to S8

Tables ST1-ST3

**Other Supplementary Material for this manuscript includes the following:**

Chemistry Protocols for synthesis and purification of Glafenine analogues

**SUPPLEMENTARY FIGURE S1.** Is the full gel version of Figure 1B showing the inability of Glafenine to affect the thermostability of CFTR in the CETSA assay (n=4).

**SUPPLEMENTARY FIGURE S2** A full gel version of Figure 1C showing the ability of Glafenine (10µM) after 24 hours treatment to correct F508del-CFTR (n=4). The complete blot several different experiments were cell were treated for 24 hours with glafenine (all 10µM) lanes 7, 8 and 9 come from the same experiment (and are used in figure 1D).

**37**

**40 10**

**53 5 7**

**24 29 34**

**27 3 1**

**30 22 38**

**18 14 2**

**4 39 54**

**11 46 8**

**56 20 25**

**26 35 16**

**19 21**

**43 17 23 52**

**45 31 6 15**

**32 50 13 42**

**47 49 48 51**

**12 9 41 28**

**33 44 55**

**36**

**SUPPLEMENTARY FIGURE S3.** Chemical structures of all the Glafenine analogues tested in this paper and their compound number.

**SUPPLEMENTARY FIGURE S4.** Is the full gel version of Figure 2D demonstrating the ability of certain Glafenine derivatives (10µM) to correct F508del-CFTR after 24 hours treatment (n=4).

**SUPPLEMENTARY FIGURE S5.** Is the full gel version of Figure 4C showing the effect of siRNA to COX1 and COX2 on their protein expression.

**SUPPLEMENTARY FIGURE 6.** This is an FMP assay performed in HEK cells to test the ability of prostaglandins PGD2 and PGE2 to inhibit the functional trafficking of F508del-CFTR mediated by glafenine (10µM) or Trikafta. Cell were treated for 24 hours with either glafenine or trikafta both with or without either PGD2 or PGE2 (both at 10 µM) and then assayed. Results clearly show that exogenous PGE2 inhibits glafenine’s ability to rescue CFTR whereas PGD2 does not and the presence of neither prostaglandin affects Trikafta mediated CFTR rescue.

**SUPPLEMENTARY FIGURE S7.** Is an FMP assay that demonstrates the inability of Prostaglandin E2 (PGE2) over a range of concentrations to disrupt the ability of VX-809 (3µM) to functionally correct 508del-CFTR. Both the VX-809 and the PGE2 were added 24 hours prior to the assay. This experiment in conjunction with that shown in Figure 6A and shows that the disruptive effect of PGE2 to Glafenine CFTR rescue is not universal to all CFTR correctors.

**SUPPLEMENTARY FIGURE S8.** Representation of the ability of Glafenine, MF63 and compound 49 to correct G85E/621-1GT heterozygote CFTR as a percentage of wild-type CFTR function. Functional expression in well-differentiated primary human bronchial epithelial (HBE) cells determined from the increase in short-circuit current stimulated by acute addition of forskolin + genistein (Δ*I*sc). Representative I_sc_ responses of primary HBE cells expressing G85E/621-1GT-CFTR to sequential addition of 10*µ*M forskolin, 50*µ*M genistein, and 10*µ*M CFTRinh-172 after 24 h preincubation with 0.1% dimethylsulfoxide (vehicle), VX-809 (1μM), Glafenine, compound 49, and MF63 (all at 10*µ*M). Data presented as means ± SEM, n = 4, *, p < 0.05, **, *p<*0.01 and ***, *p<*0.001.

| **NSAID** | **% Surface CFTR** | **NSAID** | **% Surface CFTR** |
| --- | --- | --- | --- |
| **Salicylates** |  | **2-Arylpropionic acids** |  |
| **Aspirin** | **1** | **Ibuprofen** | **25.5** |
| **Diflunisal** | **2** | **Carprofen** | **19** |
| **Methyl salicylate** | **1** | **Fenbufen** | **14.5** |
| **Magnesium Salicylate** | **0** | **Fenoprofen** | **16** |
| **Salicylic salicylate** | **3** | **Flurbiprofen** | **17** |
| **Aryl alkanoic acids** |  | **Ketoprofen** | **15** |
| **Diclofenac** | **16.5** | **Loxoprofen** | **3.5** |
| **Aceclofenac** | **2** | **Naproxen** | **15.5** |
| **Acemetacin** | **18.5** | **Tiaprofenic acid** | **3** |
| **Etodolac** | **15** | **Suprofen** | **2** |
| **Indomethacin** | **5** | **N-Aryl anthranilic acids** |  |
| **Ketorolac** | **23** | **Glafenine** | **27.5** |
| **Nabumetone** | **15.5** | **Mefenamic acid** | **15** |
| **Sulindac** | **16** | **COX-2 Inhibitors** |  |
| **Tolmetin** | **15** | **Celecoxib** | **23** |
| **Pyrazolidine derivatives** |  | **Rofecoxib** | **17.5** |
| **Phenylbutazone** | **5** | **Valdecoxib** | **14** |
| **Azapropazone** | **16** | **Sulphonanilides** |  |
| **Metamizole** | **2.5** | **Nimesulide** | **21** |
| **Oxyphenbutazone** | **4** |  |  |
| **Oxicams** |  |  |  |
| **Piroxicam** | **4** |  |  |
| **Lornoxicam** | **6** |  |  |
| **Meloxicam** | **18.5** |  |  |
| **Tenoxicam** | **21.5** |  |  |

**SUPPLEMENTARY TABLE 1 ST1.**  Table shows the names of the various NSAID compounds that were tested for their ability to correct F508del-CFTR in the surface expression HTS assay and the responses after (10µM) treatment for 24 hours expressed as a percentage of wild-type CFTR surface expression. Note any response over 10% of wild-type signal is considered a positive response (n=4).

| **ID** | **E_MAX_ (µM)** | **EC_50_ (µM)** |
| --- | --- | --- |
| **49** | 6.9 | 0.15 |
| **53** | 9.6 | 1.12 |
| **54** | 7.2 | 0.34 |
| **55** | 1.4 | 0.04 |
| **56** | 5.9 | 0.91 |

**SUPPLEMENTARY TABLE 2 ST2.** Gives the dose that gives the maximum effect (E_MAX_) and the dose that gives 50% of the maximum dose (EC_50_) as measured in FMP assay in BHK cells expressing F508del-CFTR. Related to the results in figure 2B.

**General Experimental Information**

**Chemicals**

Unless otherwise stated, all solvents and reagents were obtained from commercial suppliers, or GSK’s internal compound collection, and used without further purification. The commercially available drugs Glafenine **38** and chloroquine **42** were not prepared at GSK, and are not included in this experimental. 3,3-Dimethyl-2-oxobutyl 2-oxo-2,3-dihydroquinoline-4-carboxylate **26** was purchased from Enamine and was used as received.

**Chromatography**

Thin layer chromatography (TLC) was carried out with plastic-backed 50 precoated silica plates as the stationary phase (particle size 0.2 mm). Spots were visualized by ultraviolet (UV) light (λ max = 254 nm or 365 nm) in all cases. Normal phase silica gel chromatography was carried out using a Jones Chromatography FlashMaster Automated Flash Chromatography System, employing commercially available pre-packed SiO_2_ cartridges.

**Mass Directed Auto Prep**

Mass Directed Auto Prep purification was performed on an Agilent® 1260 Infinity II UPLCTM instrument using a SUNFIRE® or ATLANTIS® C18 column (19 ᵡ 100 mm, 5.0 μm packing diameter) and either an Agilent® Infinity lab LC/MSD TM or Agilent® 6120 single quadrupole LC/MSTM machine using alternative scan positive and negative electrospray. Analytes were detected at a selected UV wavelength, generally 210, 230 or 254 nm.

Gradient elution occurred at ambient temperature with the eluents as (A) H_2_O containing 0.1% volume/volume (v/v) formic acid and (B) acetonitrile containing 0.1% (v/v) formic acid.

The elution gradients used were at a flow rate of 20 mL/min over 20 or 30 min, and either standard or focused purification methods were used as required.

| Standard Methods | Composition | Focused Methods | Composition |
| --- | --- | --- | --- |
| Method A | 20-32.5% B | Focused Method A | 15-37.5% B |
| Method B | 32.5-45% B | Focused Method B | 30-55% B |
| Method C | 45-58% B | Focused Method C | 40-65% B |
| Method D | 58-70% B | Focused Method D | 50-75% B |
| Method E | 70-100% B | Focused Method E | 60-85% B |
| Method F | N/a | Focused Method F | 75-100% B |

**Liquid Chromatography Mass Spectrometry (LCMS)**

LCMS analysis was performed on an Acquity^®^ UPLC instrument. Mass spectra were recorded with a Waters ZQ spectrometer using alternate-scan positive and negative electrospray.

Two methods were used for LCMS, as described below.

**System A:**

Samples were eluted through a BEH C18 column (50 mm x 2.1 mm, 1.7 μm packing diameter), at 40 °C. The solvents employed were:

A: 0.1% v/v formic acid in water

B: 0.1% v/v formic acid in acetonitrile.

The gradient employed was:

| **Time (min)** | **Flow rate (mL/min)** | **%A** | **%B** |
| --- | --- | --- | --- |
| 0 | 1 | 97 | 3 |
| 1.5 | 1 | 0 | 100 |
| 1.9 | 1 | 0 | 100 |
| 2.0 | 1 | 97 | 3 |

**System B:**

Samples were eluted through a Sunfire C18 column (30 mm x 4.6 mm, 3.5 μm packing diameter), at 30 °C. The solvents employed were:

A: 0.1% v/v formic acid in water

B: 0.1% v/v formic acid in acetonitrile.

The gradient employed was:

| **Time (min)** | **Flow rate (mL/min)** | **%A** | **%B** |
| --- | --- | --- | --- |
| 0 | 3 | 97 | 3 |
| 0.1 | 3 | 97 | 3 |
| 4.2 | 3 | 0 | 100 |
| 4.8 | 3 | 0 | 100 |
| 4.9 | 3 | 97 | 3 |
| 5.0 | 3 | 97 | 3 |

**Nuclear Magnetic Resonance (NMR) Spectroscopy**

^1^H NMR spectra were recorded in commercially supplied deuterated solvents at ambient temperature using standard pulse methods with the following spectrometers, and associated signal frequencies: Bruker^®^ AV-400^TM^ (^1^H = 400 MHz) and Bruker^®^ AV-600 ^TM^ (^1^H = 600 MHz). Chemical shifts (δ) are reported in ppm and are relative to tetramethylsilane (TMS) reference, where δ (TMS) = 0.00 ppm, or the peaks corresponding to trace non-deuterated solvent. All NMR analysis were performed in commercially supplied deuterated solvents chloroform-*d*, methanol-*d*_4_ and dimethylsulfoxide‑*d*_6_. Coupling constants (*J*) are reported to the nearest 0.1 Hz for ^1^H NMR, and the multiplicities of signals are described as singlet (s), doublet (d), triplet (t), quartet (q), quintet (quin), sextet (sxt), broad (br) and multiplet (m), or a combination of these descriptors for extended coupling patterns. The number of hydrogen responsible for a signal is indicated by nH.

**Experimental protocols Compound Synthesis:**

**Preparation of 2-((7-chloroquinolin-4-yl)amino)-N-(3-hydroxypropyl)benzamide, 1**

**

HATU (4.8 mmol) was dissolved in 9.6 mL DMF, and added to 2-[(7-chloro-4-quinolinyl)amino]benzoic acid (720mg, 2.4mmol). To this solution was added DIPEA (1.25 mL, 7.2 mmol), and the resulting solution was shaking for 5 minutes. 0.452 mL of the solution was added to the pre-weighed amine (0.1 mmol) in 0.2 mL DMF, the solution shaken for 10 minutes, and left overnight at room temperature. The sample was purified by Mass Directed AutoPrep (Sunfire C18 column, MeCN/H_2_O with formic acid modifier), and the solvent removed under a stream of nitrogen to afford 2-((7-chloroquinolin-4-yl)amino)-*N*-(3-hydroxypropyl)benzamide (4.8 mg, 12% yield).

**LCMS** (system A): t_ret_ 0.55 min, MH+ 356/358

**^1^H NMR** (400 MHz, DMSO-d_6_): δ ppm 10.89 (br s, 1H), 8.69-8.84 (m, 1H), 8.56 (d, *J* = 5.0 Hz, 1H), 8.14 (d, *J* = 9.1 Hz, 1H), 7.96 (s, 1H), 7.77 (d, *J* = 7.6 Hz, 1H), 7.67 (d, *J* = 8.6 Hz, 2H), 7.53 (t, *J* = 7.3 Hz, 1H), 7.14-7.23 (m, 2H), 3.45-3.22 (m, 4H), 1.52-1.67 ppm (m, 2H)

**Preparation of (*S*)-(2-((7-chloroquinolin-4-yl)amino)phenyl)(2-(hydroxymethyl)pyrrolidin-1-yl)methanone, 2**

**

HATU (4.8 mmol) was dissolved in 9.6 mL DMF, and added to 2-[(7-chloro-4-quinolinyl)amino]benzoic acid (720mg, 2.4mmol). To this solution was added DIPEA (1.25 mL, 7.2 mmol), and the resulting solution was shaking for 5 minutes. 0.452 mL of the solution (0.1 mmol acid, 0.2 mmol HATU, 0.3 mmol DIPEA) was added to the pre-weighed amine (0.1 mmol) in 0.2 mL DMF, the solution shaken for 10 minutes, and left overnight at room temperature. The sample was purified by Mass Directed AutoPrep (Sunfire C18 column, MeCN/H_2_O with formic acid modifier), and the solvent removed under a stream of nitrogen to afford (*S*)-(2-((7-chloroquinolin-4-yl)amino)phenyl)(2-(hydroxymethyl)pyrrolidin-1-yl)methanone (8.5 mg, 20% yield).

**LCMS** (system A): t_ret_ 0.60 min, MH+ 382/384

**^1^H NMR** (400 MHz, DMSO-d_6_) δ ppm 8.36 (1H, d, *J* = 5.5 Hz), 8.34-8.29 (1H, m), 8.19 (1H, s), 7.90-7.86 (1H, m), 7.71-7.39 (4H, m), 7.35-7.28 (1H, m), 6.56-6.48 (1H, m), 3.86-3.77 (1H, m), 3.38-3.28 (1H, m), 3.24-3.15 (1H, m), 3.12-2.99 (2H, m), 2.65-2.57 (1H, m), 1.77-1.53 (4H, m)

**Preparation of (*R*)-2-((7-chloroquinolin-4-yl)amino)-*N*-(1-hydroxypropan-2-yl)benzamide, 3**

**

HATU (4.8 mmol) was dissolved in 9.6 mL DMF, and added to 2-[(7-chloro-4-quinolinyl)amino]benzoic acid (720mg, 2.4mmol). To this solution was added DIPEA (1.25 mL, 7.2 mmol), and the resulting solution was shakem for 5 minutes. 0.452 mL of the solution was added to the pre-weighed amine (0.1 mmol) in 0.2 mL DMF, the solution shaken for 10 minutes, and left overnight at room temperature. The sample was purified by Mass Directed AutoPrep (Sunfire C18 column, MeCN/H_2_O with formic acid modifier), and the solvent removed under a stream of nitrogen to afford (*R*)-2-((7-chloroquinolin-4-yl)amino)-*N*-(1-hydroxypropan-2-yl)benzamide (4.0 mg, 10% yield)

**LCMS** (system A): t_ret_ 0.56 min, MH+ 356/358

**^1^H NMR** (400 MHz, DMSO-d_6_) δ ppm 10.74 (br s, 1H), 8.55 (d, *J* = 4.5 Hz, 1H), 8.45-8.33 (m, 1H), 8.16 (d, *J* = 8.6 Hz, 1H), 7.95 (br. s, 1H), 7.80 (d, *J* = 7.6 Hz, 1H), 7.65 (t, J 8.1 Hz, 2H), 7.54 (t, *J* = 7.6 Hz, 1H), 7.18 (t, *J* = 7.6 Hz, 1H), 7.15-7.08 (m, 1H), 4.87-4.61 (br s, 1H), 4.00-3.88 (m, 1H), 3.45-3.11 (m, 2H) 3.28-3.19 (m, 1H), 1.01 (d, *J* = 6.6 Hz, 3H)

**Preparation of (3-methyloxetan-3-yl)methyl 2-((7-chloroquinolin-4-yl)amino)benzoate, 4**

**

To a vial containing 2-[(7-chloro-4-quinolinyl)amino]benzoic acid (0.1 mmol) in 0.6 mL DMF was added the alcohol (0.3 mmol), a heaped microspatula of PyBOP, and DIPEA (0.052 mL, 0.3 mmol). The resultant solution was shaken for 10 minutes, and left overnight at room temperature. The sample was purified by Mass Directed Auto Prep (Sunfire C18 column, MeCN/H_2_O with formic acid modifier), and the solvent removed under a stream of nitrogen to afford (3-methyloxetan-3-yl)methyl 2-((7-chloroquinolin-4-yl)amino)benzoate (7.4 mg, 17% yield).

**LCMS** (system A): t_ret_ 0.73 min, MH+ 383/385

**^1^H NMR** (400 MHz, CD_3_OD) δ ppm 8.53 (d, *J* = 5.5 Hz, 1H), 8.21 (d, *J* = 9.1 Hz, 1H), 8.15 (dd, *J* = 8.1, 1.5 Hz, 1H), 7.93 (d, *J* = 2.0 Hz, 1H), 7.70-7.75 (m, 1H), 7.62-7.69 (m, 1H), 7.60 (dd, *J* = 9.1, 2.0 Hz, 1H), 7.30 (d, *J* = 5.5 Hz, 1H), 7.18 - 7.26 (m, 1H), 4.60 (d, *J* = 6.0 Hz, 2H), 4.39-4.43 (m, 4H), 1.38 (s, 3H)

**Methyl 2-((7-chloroquinolin-4-yl)amino)benzoate, 5**

**

Methyl 2-((7-chloroquinolin-4-yl)amino)benzoate was ordered from GSK solid stores. The structure and purity were confirmed by NMR.

**LCMS** (system A): t_ret_ 0.70 min, MH+ 313/315

**^1^H NMR** (400 MHz, CDCl_3_) δ ppm 11.42 (br s, 1H), 8.63-8.74 (m, 1H), 8.54 (d, *J* = 6.5 Hz, 1H), 8.12-8.23 (m, 2H), 7.65-7.83 (m, 3H), 7.49 (d, *J* = 6.5 Hz, 1H), 7.36 (br d, *J* = 7.1 Hz, 1H), 4.01 ppm (s, 3H)

**Preparation of (2-((5-chloropyrazolo[1,5-a]pyrimidin-7-yl)amino)phenyl)(4-methylpiperazin-1-yl)methanone, 6**

**

The carboxylic acid (0.176 mmol) was dissolved in DMF (0.8 mL), and added to a solution of HATU (0.35 mmol) in DMF. DIPEA (0.524 mmol) was added, and the solution shaken for 10 minutes at room temperature. 0.33 mL of the solution was dispensed into a vial, and amine (0.44 mmol) was added, the solution shaken for 10 minutes, and the reaction left overnight at room temperature. The reaction mixture was loaded onto a 1g aminopropyl SPE cartridge (pre-washed with 2 x 3 mL MeOH, and 6 mL chloroform), left for 1 hour to absorb, and eluted with 10% MeOH in EtOAC (2 x 3 mL), and the solvent removed *in vacuo*. The sample was dissolved in DMSO (0.5 mL) and purified by Mass Directed Auto Prep (Sunfire C18 column, MeCN/H_2_O with formic acid modifier), and the solvent removed under a stream of nitrogen to afford 2-((5-chloropyrazolo[1,5-a]pyrimidin-7-yl)amino)phenyl)(4-methylpiperazin-1-yl)methanone (1.3 mg, 7% yield).

**LCMS** (system A): t_ret_ 0.62 min, MH+ 371/373

**^1^H NMR** (400 MHz, CDCl_3_) δ ppm 9.42 (br s, 1H), 8.11 (d, *J* = 2.5 Hz, 1H), 7.68 (d, *J* = 8.1 Hz, 1H), 7.55 (td, *J* = 7.8, 1.5 Hz, 1H), 7.41 (dd, *J* = 7.6, 1.5 Hz, 1H), 7.27-7.37 (m, 1H), 6.52 (d, *J* = 2.0 Hz, 1H0, 6.47 (s, 1H), 4.04-4.14 (m, 1H), 3.73-3.94 (m, 2H), 3.45-3.60 (m, 2H), 2.30-2.52 (m, 4H), 2.27 (s, 3H)

**Preparation of 2-((7-chloroquinolin-4-yl)amino)-*N*-(2,3-dihydroxypropyl)benzamide, 7**

**

1-(2,2-Dimethyl-1,3-dioxolan-4-yl)methanamine (0.016 g, 0.12 mmol) was weighed into tubes, followed by triazabicyclo[4.4.0]dec-5-ene (4.2 mg, 0.03 mmol). A solution of ester (0.1 mmol) in THF (0.6 mL) was dispensed into the tube, and the solution stirred at 75 ^o^C for 12 hours, and the solvent was removed under a stream of nitrogen. The sample was redissolved in DMSO (0.5 mL), and purified by Mass Directed Auto Prep (Sunfire C18 column, MeCN/H_2_O with formic acid modifier), and the solvent removed under a stream of nitrogen to afford 2-((7-chloroquinolin-4-yl)amino)-*N*-(2,3-dihydroxypropyl)benzamide (15.4 mg, 41%).

**LCMS** (system A): t_ret_ 0.53 min, MH+ 372/374

**^1^H NMR** (400 MHz, CD_3_OD) δ ppm 8.51 (d, *J* = 9.6 Hz, 1H), 8.41 (d, *J* = 7.1 Hz, 1H), 7.97 (d, *J* = 2.0 Hz, 1H), 7.80-7.86 (m, 2H), 7.70-7.75 (m, 1H), 7.64-7.68 (m, 1H), 7.56 (td, *J* = 7.6, 1.5 Hz, 1H), 6.88 (d, *J* = 7.1 Hz, 1H), 3.60-3.75 (m, 1H), 3.39-3.50 (m, 3H), 3.32-3.38 (m, 1H)

**Preparation of (tetrahydrofuran-3-yl)methyl 2-((7-chloroquinolin-4-yl)amino)benzoate, 8**

**

To a vial containing 2-[(7-chloro-4-quinolinyl)amino]benzoic acid (0.1 mmol) in 0.6 mL DMF was added the alcohol (0.3 mmol), a heaped microspatula of PyBOP, and DIPEA (0.052 mL, 0.3 mmol). The resultant solution was shaken for 10 minutes, and left overnight at room temperature. The sample was purified by Mass Directed Auto Prep (Sunfire C18 column, MeCN/H_2_O with formic acid modifier), and the solvent removed under a stream of nitrogen to afford (tetrahydrofuran-3-yl)methyl 2-((7-chloroquinolin-4-yl)amino)benzoate (16.5 mg, 39%).

**LCMS** (system A): t_ret_ 0.75 min, MH+ 383/385

**^1^H NMR** (400 MHz, CD_3_OD) δ ppm 8.53 (d, *J* = 5.5 Hz, 1H), 8.20 (d, *J* = 9.1 Hz, 1H), 8.10 (dd, *J* = 8.1, 1.5 Hz, 1H), 7.92 (d, *J* = 2.0 Hz, 1H), 7.69-7.74 (m, 1H), 7.57-7.66 (m, 2H), 7.31 (d, *J* = 5.5 Hz, 1H), 7.16-7.23 (m, 1H), 4.20-4.36 (m, 2H), 3.80-3.90 (m, 2H), 3.69-3.77 (m, 1H), 3.59-3.66 (m, 1H), 2.65-2.74 (m, 1H), 2.03-2.14 (m, 1H), 1.65-1.78 ppm (m, 1H)

**Preparation of methyl 4-amino-5-(7-chloro-4-quinolinyl)-3-thiophenecarboxylate, 9**

**

To a microwave vial containing methyl 4-amino-3-thiophenecarboxylate hydrochloride (196 mg, 1.01 mmol) and 4.7-dichloroquinoline (200 mg, 1.01 mmol) was added isopropanol (4 mL), and 2 M HCl (0.4 mL) was heated to 140 ^o^C in a microwave reactor. The reaction mixture was diluted with water, then extracted three times with EtOAc, and the combined organic layers washed three times with brine, dried over magnesium sulfate, filtered, and the solvent removed *in vacuo*. The crude sample was purified by flash column chromatography (SiO_2_, 50g) eluting with 0-100% EtOAc in cyclohexane, and the solvent removed *in vacuo* to give two Regio isomers. The mixture was dissolved in DMSO and purified by Mass Directed Auto Prep (Sunfire C18 column, MeCN/H_2_O with formic acid modifier), and the solvent removed under a stream of nitrogen to afford methyl 4-amino-5-(7-chloro-4-quinolinyl)-3-thiophenecarboxylate (3.3 mg, 1% yield).

**LCMS** (system A): t_ret_ 2.77 min, MH+ 319/321

**^1^H NMR** (600 MHz, CDCl_3_) δ ppm 8.93 (d, *J* = 4.4 Hz, 1H), 8.25 (br s, 1H), 8.20 (s, 1H), 8.00 (d, *J* = 8.8 Hz, 1H), 7.57 (dd, *J* = 9.0, 2.0 Hz, 1H), 7.48 (d, *J* = 4.8 Hz, 1H), 4.96-5.09 (m, 2H), 3.93 ppm (s, 3H)

**Preparation of (4-aminopiperidin-1-yl)(2-(quinolin-4-ylamino)phenyl)methanone, 10**

**

The carboxylic acid (0.72 mmol) was dissolved in DMF (3 mL), added to a solution of HATU (1.44 mmol) and DIPEA (2.16 mmol) in DMF (1.8 mL, and the solution shaken for 10 minutes at room temperature. 0.86 mL of the solution was dispensed into a vial, amine (0.12 mmol) was added, the solution shaken for 10 minutes, and the reaction left overnight at room temperature. The reaction mixture was loaded onto a 1g aminopropyl SPE cartridge (pre-washed with 2 x 3 mL MeOH, and 6 mL chloroform), left for 1 hour to absorb, and eluted with 10% MeOH in EtOAC (2 x 3 mL), and the solvent removed *in vacuo*. The sample was dissolved in DMSO (0.5 mL) and purified by Mass Directed Auto Prep (Sunfire C18 column, MeCN/H_2_O with formic acid modifier), and the solvent removed under a stream of nitrogen to afford (4-aminopiperidin-1-yl)(2-(quinolin-4-ylamino)phenyl)methanone (17.4 mg, 38%).

**LCMS** (system A): t_ret_ 0.36 min, MH+ 347

**^1^H NMR** (400 MHz, DMSO-d_6_) δ ppm 10.76 (s, 1H), 8.67 (d, *J* = 8.6 Hz, 1H), 8.51 (d, *J* = 6.5 Hz, 1H), 7.98-8.08 (m, 3H), 7.81 (ddd, *J* = 8.4, 6.2, 2.0 Hz, 1H), 7.66-7.72 (m, 1H), 7.58-7.64 (m, 1H), 7.50-7.57 (m, 2H), 6.43 (d, *J* = 7.1 Hz, 1H), 4.17-4.32 (m, 1H), 3.47-3.59 (m, 1H), 3.16-3.33 (m, 1H), 2.92-3.12 (m, 1H), 2.81-2.92 (m, 1H), 1.74-1.93 (m, 2H), 1.10-1.57 ppm (m, 2H)

**Preparation of 6(2-((7-chloroquinolin-4-yl)amino)phenyl)(4-hydroxypiperidin-1-yl)methanone, 11**

**

Methyl 2-((7-chloroquinolin-4-yl)amino)benzoate (0.6 mmol) was dissolved in THF (6 mL) and 1ml (0.1mmol) dispensed into a vial containing pre-weighed amines (0.15mmol). Triazabicyclo[4.4.0]dec-5-ene (0.03 mmol) was added to each vial, and the vials sealed and heated in a CEM Discover microwave reactor using initial power 150 W to 140 ^o^C for 10 minutes. The samples were dissolved in DMSO (0.5 mL), and purified by Mass Directed Auto Prep (Sunfire C18 column, MeCN/H_2_O with formic acid modifier), and the solvent removed under a stream of nitrogen to afford (2-((7-chloroquinolin-4-yl)amino)phenyl)(4-hydroxypiperidin-1-yl)methanone (0.5 mg, 1% yield).

**LCMS** (system A): t_ret_ 0.52 min, MH+ 382/384

**^1^H NMR** (400 MHz, CDCl_3_) δ ppm 8.74-8.92 (m, 1H), 8.62 (d, *J* = 5.5 Hz, 1H), 8.04-8.10 (m, 1H), 7.93 (d, *J* = 9.1 Hz, 1H), 7.65-7.72 (m, 1H), 7.43-7.54 (m, 2H), 7.33-7.39 (m, 1H), 7.14-7.21 (m, 2H), 3.75-4.19 (m, 3H), 3.29-3.55 (m, 2H), 1.76-2.01 (m, 2H), 1.42-1.65 ppm (m, 2H)

**Preparation of *N*-(3-(1*H*-tetrazol-5-yl)phenyl)quinolin-4-amine, 12**

**

To a microwave vial containing methyl 3-(1*H*-tetrazol-5-yl)aniline (100 mg, 0.620 mmol) and 4‑chloroquinoline (102 mg, 0.620 mmol) was added isopropanol (4 mL), and 2 M HCl (0.31 mL), and the reaction heated to 140 ^o^C in a microwave reactor. The reaction mixture was diluted with 2M NaOH, and the aqueous phase washed with EtOAc (10 mL). The basic aqueous phase was acidified with 1M HCl, upon which product was observed to precipitate. The solid was filtered, washed with water, and dried *in vacuo* to afford *N*-(3-(1*H*-tetrazol-5-yl)phenyl)quinolin-4-amine (80.4 mg, 45% yield).

**LCMS** (system A): t_ret_ 1.19 min, MH+ 289

**^1^H NMR** (400 MHz, DMSO-d_6_) δ ppm 15.80 (br s, 1H), 11.21 (s, 1H), 8.89 (d, *J* = 8.3 Hz, 1H), 8.58 (d, *J* = 7.1 Hz, 1H), 8.25 (t, *J*= 1.6 Hz, 1H), 8.10-8.19 (m, 2H), 8.02-8.10 (m, 1H), 7.77-7.89 (m, 2H), 7.70-7.77 (m, 1H), 6.98 (d, *J* = 7.1 Hz, 1H)

**Preparation of *N*-[3-(1*H*-tetrazol-5-yl)phenyl]thieno[3,2-b]pyridin-7-amine, 13**

**

To a microwave vial containing methyl 3-(1*H*-tetrazol-5-yl)aniline (100 mg, 0.620 mmol) and 7-chlorothieno[3,2-b]pyridine (105 mg, 0.620 mmol) was added isopropanol (4 mL), and 2 M HCl (0.31 mL), and the reaction heated to 140 ^o^C in a microwave reactor. The reaction mixture was diluted with 2M NaOH, and the aqueous phase washed with EtOAc (10 mL). The basic aqueous phase was acidified with 1M HCl, upon which product was observed to precipitate. The solid was filtered, washed with water, and dried *in vacuo* to afford *N*-[3-(1*H*-tetrazol-5-yl)phenyl]thieno[3,2-b]pyridin-7-amine (162 mg, 89%)

**LCMS** (system A): t_ret_ 1.16 min, MH+ 295

**^1^H NMR** (400 MHz, DMSO-d_6_) δ ppm 10.93 (s, 1H), 8.48 (d, *J* = 7.1 Hz, 1H), 8.43 (d, *J* = 5.5 Hz, 1H), 8.13-8.16 (m, 1H), 8.07-8.12 (m, 1H), 7.78 (t, *J* = 8.1 Hz, 1H), 7.66-7.71 (m, 1H), 7.63 (d, *J* = 5.5 Hz, 1H), 7.07 ppm (d, *J* = 6.5 Hz, 1H)

**Preparation of *N*-(2-acetamidoethyl)-2-((7-chloroquinolin-4-yl)amino)benzamide, 14**

**

HATU (4.8 mmol) was dissolved in 9.6 mL DMF, and added to 2-[(7-chloro-4-quinolinyl)amino]benzoic acid (720mg, 2.4mmol). To this solution was added DIPEA (1.25 mL, 7.2 mmol), and the resulting solution was shaken for 5 minutes. 0.452 mL of the solution (0.1 mmol acid, 0.2 mmol HATU, 0.3 mmol DIPEA) was added to the pre-weighed amine (0.1 mmol) in 0.2 mL DMF, the solution shaken for 10 minutes, and left overnight at room temperature. The sample was purified by Mass Directed AutoPrep (Sunfire C18 column, MeCN/H_2_O with formic acid modifier), and the solvent removed under a stream of nitrogen to afford *N*-(2-acetamidoethyl)-2-((7-chloroquinolin-4-yl)amino)benzamide (5.7 mg, 13% yield).

**LCMS** (system A): t_ret_ 0.55 min, MH+ 383/385

**^1^H NMR** (400 MHz, DMSO-d_6_) δ ppm 10.85 (br s, 1H), 8.78-8.86 (m, 1H), 8.52-8.59 (m, 1H), 8.11-8.17 (m, 1H), 7.93-8.00 (m, 2H), 7.78 (dd, *J* = 7.8, 1.3 Hz, 1H), 7.66 (dd, J = 8.8, 2.3 Hz, 2H), 7.51-7.57 (m, 1H), 7.13-7.22 (m, 2H), 3.22-3.32 (m, 2H), 3.10-3.18 (m, 2H), 1.74 ppm (s, 3H)

**Preparation of *N*-(2,3-dihydroxypropyl)-2-(quinolin-4-ylamino)benzamide, 15**

**

The carboxylic acid (0.72 mmol) was dissolved in DMF (3 mL), added to a solution of HATU (1.44 mmol) and DIPEA (2.16 mmol) in DMF (1.8 mL), and the solution shaken for 10 minutes at room temperature. 0.86 mL of the solution was dispensed into a vial, amine (0.12 mmol) was added, the solution shaken for 10 minutes, and the reaction left overnight at room temperature. The reaction mixture was loaded onto a 1g aminopropyl SPE cartridge (pre-washed with 2 x 3 mL MeOH, and 6 mL chloroform), left for 1 hour to absorb, and eluted with 10% MeOH in EtOAC (2 x 3 mL), and the solvent removed *in vacuo*. The sample was dissolved in DMSO (0.5 mL) and purified by Mass Directed Auto Prep (Sunfire C18 column, MeCN/H_2_O with formic acid modifier), and the solvent removed under a stream of nitrogen to afford *N*-(2,3-dihydroxypropyl)-2-(quinolin-4-ylamino)benzamide (14.3 mg, 32% yield).

**LCMS** (system A): t_ret_ 0.44 min, MH+ 338

**^1^H NMR** (400 MHz, DMSO-d_6_) δ ppm 11.29 (br s, 1H), 8.70 (d, *J* = 8.6 Hz, 1H), 8.65 (t, *J* = 5.5 Hz, 1H), 8.50 (*d*, J = 6.5 Hz, 1H), 7.99-8.13 (m, 2H), 7.77-7.86 (m, 2H), 7.64-7.71 (m, 1H), 7.50-7.61 (m, 2H), 6.60 (*d*, J = 6.5 Hz, 1H), 3.41-3.48 (m, 1H), 3.15-3.28 (m, 3H), 2.98-3.07 ppm (m, 1H)

**Preparation of 4-aminopiperidin-1-yl)(2-((7-chloroquinolin-4-yl)amino)phenyl)methanone, 16**

**

The carboxylic acid (1.2 mmol) was dissolved in DMF (1.8 mL), added to a solution of HATU (1.2 mmol) and DIPEA (1.8 mmol) in DMF (1.8 mL), and the solution shaken for 10 minutes at room temperature. 0.65 mL of the solution was dispensed into a vial, amine (0.10 mmol) was added, the solution shaken for 10 minutes, and the reaction left overnight at room temperature. The reaction mixture was loaded onto a 1g aminopropyl SPE cartridge (pre-washed with 2 x 3 mL MeOH, and 6 mL chloroform), left for 1 hour to absorb, and eluted with 10% MeOH in EtOAC (2 x 3 mL), and the solvent removed *in vacuo*. The sample was dissolved in DMSO (0.5 mL) and purified by Mass Directed Auto Prep (Sunfire C18 column, MeCN/H_2_O with formic acid modifier), and the solvent removed under a stream of nitrogen to afford (4-aminopiperidin-1-yl)(2-((7-chloroquinolin-4-yl)amino)phenyl)methanone (15.6 mg, 37% yield).

**LCMS** (system A): t_ret_ 0.45 min, MH+ 381/383

**^1^H NMR** (400 MHz, CD_3_OD) δ ppm 8.52 (d, *J* = 9.1 Hz, 1H), 8.39 (d, *J* = 7.1 Hz, 1H), 7.99 (d, *J* = 2.0 Hz, 1H), 7.81 (dd, *J* = 9.1, 2.0 Hz, 1H), 7.70-7.76 (m, 1H), 7.65 (td, *J* = 7.6, 1.0 Hz, 1H), 7.56-7.61 (m, 2H), 6.58 (d, *J* = 7.1 Hz, 1H), 4.46-4.57 (m, 1H), 3.68-3.80 (m, 1H), 3.32-3.40 (m, 2H), 3.10-3.26 (m, 1H), 1.94-2.09 (m, 2H), 1.41-1.59 ppm (m, 2H)

**Preparation of 2-((5-chloropyrazolo[1,5-a]pyrimidin-7-yl)amino)-*N*-(2,3-dihydroxypropyl)benzamide, 17**

**

The carboxylic acid (0.176 mmol) was dissolved in DMF (0.8 mL), and added to a solution of HATU (0.35 mmol) in DMF. DIPEA (0.524 mmol) was added, and the solution shaken for 10 minutes at room temperature. 0.33 mL of the solution was dispensed into a vial, and amine (0.44 mmol) was added, the solution shaken for 10 minutes, and the reaction left overnight at room temperature. The reaction mixture was loaded onto a 1g aminopropyl SPE cartridge (pre-washed with 2 x 3 mL MeOH, and 6 mL chloroform), left for 1 hour to absorb, and eluted with 10% MeOH in EtOAC (2 x 3 mL), and the solvent removed *in vacuo*. The sample was dissolved in DMSO (0.5 mL) and purified by Mass Directed Auto Prep (Sunfire C18 column, MeCN/H_2_O with formic acid modifier), and the solvent removed under a stream of nitrogen to afford 2-((5-chloropyrazolo[1,5-a]pyrimidin-7-yl)amino)-*N*-(2,3-dihydroxypropyl)benzamide (1.4 mg, 8% yield).

**LCMS** (system A): t_ret_ 0.75 min, MH+ 362/364

**^1^H NMR** (400 MHz, DMSO-d) δ ppm 8.06-8.19 (m, 1H), 7.89 (d, *J* = 7.6 Hz, 1H), 7.60-7.70 (m, 1H), 7.50-7.59 (m, 1H), 7.17-7.27 (m, 1H), 6.26-6.49 (m, 2H), 4.95 (br s, 1H), 4.62 (br s, 1H), 3.56-3.65 (m, 1H), 3.23-3.47 (m, 2H), 3.11-3.21 ppm (m, 2H)

**Preparation of (2-((7-chloroquinolin-4-yl)amino)phenyl)(3-(dimethylamino)azetidin-1-yl)methanone, 18**

**

HATU (4.8 mmol) was dissolved in 9.6 mL DMF, and added to 2-[(7-chloro-4-quinolinyl)amino]benzoic acid (720mg, 2.4mmol). To this solution was added DIPEA (1.25 mL, 7.2 mmol), and the resulting solution was shaking for 5 minutes. 0.452 mL of the solution (0.1 mmol acid, 0.2 mmol HATU, 0.3 mmol DIPEA) was added to the pre-weighed amine (0.1 mmol) in 0.2 mL DMF, the solution shaken for 10 minutes, and left overnight at room temperature. The sample was purified by Mass Directed AutoPrep (Sunfire C18 column, MeCN/H_2_O with formic acid modifier), and the solvent removed under a stream of nitrogen to afford (2-((7-chloroquinolin-4-yl)amino)phenyl)(3-(dimethylamino)azetidin-1-yl)methanone (4.9 mg, 12% yield).

**LCMS** (system A): t_ret_ 0.44 min, MH+ 381/383

**^1^H NMR** (400 MHz, CD_3_OD) δ ppm 8.48 (d, *J* = 5.5 Hz, 1H), 8.18 (d, *J* = 8.6 Hz, 1H), 7.91 (d, *J* = 2.5 Hz, 1H), 7.64-7.70 (m, 1H), 7.54-7.61 (m, 3H), 7.26 (td, *J* = 7.6, 1.0 Hz, 2H), 7.08 (d, *J* = 5.5 Hz, 1H), 4.30-4.38 (m, 1H), 4.05-4.15 (m, 2H), 3.86-3.94 (m, 1H), 3.07-3.16 (m, 1H), 2.14 ppm (s, 6H)

**Preparation of 3-(quinolin-4-ylamino)benzoic acid, 19**

**

1M aq. NaOH (2.00 mL) was added to a stirred solution of ethyl 3-(4-quinolinylamino)benzoate (87 mg, 0.298 mmol) in EtOH (3 mL), and the mixture stirred for 1 hour at room temperature, then 2 hours at 80 ^o^C. The reaction mixture was cooled to room temperature, and adjusted to pH2 with 6M HCl. The solvent was removed *in vacuo*, and the residue slurried in water (3 mL). The solid was filtered, washed with water, and dried. The solid was dissolved in MeCN/H_2_O/EtOH and stirred at 60 ^o^C with SiliaBond® thiol (100 mg) for 1 hour. The mixture was filtered, and the solvent removed *in vacuo* to afford 3-(quinolin-4-ylamino)benzoic acid (58 mg, 65% yield).

**LCMS** (system A): t_ret_ 0.58 min, MH+ 265

**^1^H NMR** (400 MHz, DMSO-d_6_) 10.86 (br s, 1H), 8.75 (d, *J* = 8.3 Hz, 1H), 8.55 (d, *J* = 6.8 Hz, 1H), 7.99-8.11 (m, 3H), 7.95 (dt, *J* = 7.8, 1.3 Hz, 1H), 7.73-7.87 (m, 2H), 7.65-7.73 (m, 1H), 6.89 ppm (d, *J* = 6.8 Hz, 1H)

**Preparation of 2-((7-chloroquinolin-4-yl)amino)-*N*-(2-(methylsulfonamido)ethyl)benzamide, 20**

**

HATU (4.8 mmol) was dissolved in 9.6 mL DMF, and added to 2-[(7-chloro-4-quinolinyl)amino]benzoic acid (720mg, 2.4mmol). To this solution was added DIPEA (1.25 mL, 7.2 mmol), and the resulting solution was shaking for 5 minutes. 0.452 mL of the solution (0.1 mmol acid, 0.2 mmol HATU, 0.3 mmol DIPEA) was added to the pre-weighed amine (0.1 mmol) in 0.2 mL DMF, the solution shaken for 10 minutes, and left overnight at room temperature. The sample was purified by Mass Directed AutoPrep (Sunfire C18 column, MeCN/H_2_O with formic acid modifier), and the solvent removed under a stream of nitrogen to afford 2-((7-chloroquinolin-4-yl)amino)-*N*-(2-(methylsulfonamido)ethyl)benzamide (4.1 mg, 9% yield).

**LCMS** (system A): t_ret_ 0.57 min, MH+ 419/421

**^1^H NMR** (400 MHz, DMSO-d_6_) δ ppm 10.73-10.88 (m, 1H), 8.76-8.93 (m, 1H), 8.51-8.63 (m, 1H), 8.09-8.21 (m, 1H), 7.92-8.02 (m, 1H), 7.77-7.87 (m, 1H), 7.62-7.73 (m, 2H), 7.49-7.60 (m, 1H), 7.06-7.28 (m, 3H), 3.29-3.35 (m, 2H), 2.97-3.06 (m, 2H), 2.86 ppm (s, 3H)

**Preparation of 2,3-diethenyl-N-[2-(1H-tetrazol-5-yl)phenyl]-4-pyridinamine, 21**

**

To a microwave vial containing methyl 3-(1*H*-tetrazol-5-yl)aniline (100 mg, 0.620 mmol) and 4‑chloroquinoline (102 mg, 0.620 mmol) was added isopropanol (4 mL), and 2 M HCl (1 mL), and the reaction heated to 140 ^o^C in a microwave reactor for 1 hour. The solvent was removed *in vacuo*, and the crude residue purified by flash column chromatography (SiO_2_, 100g) eluting with 0-50% MeOH in DCM. The solvent was removed *in vacuo*, and the residue was subsequently purified by Mass Directed AutoPrep (Sunfire C18 column, MeCN/H_2_O with formic acid modifier), and the solvent removed under a stream of nitrogen to afford 2,3-diethenyl-*N*-[2-(1*H*-tetrazol-5-yl)phenyl]-4-pyridinamine (5.0 mg, 3% yield).

**LCMS** (system A): t_ret_ 0.53 min, MH+ 289

**^1^H NMR** (400 MHz, CD_3_OD) δ ppm 8.69 (d, *J* = 8.6 Hz, 1H), 8.45 (br s, 1H), 8.35 (d, *J* = 7.0 Hz, 1H), 8.19 (dd, *J* = 8.1, 1.5 Hz, 1H), 7.94-8.01 (m, 1H), 7.86-7.92 (m, 1H), 7.74-7.85 (m, 2H), 7.57 (td, *J* = 7.8, 1.5 Hz, 1H), 7.46 (td, *J* = 7.6, 1.0 Hz, 1H), 7.12 ppm (d, *J* = 6.5 Hz, 1H)

**Preparation of 2-(dimethylamino)ethyl 2-((7-chloroquinolin-4-yl)amino)benzoate, 22**

**

To a vial containing 2-[(7-chloro-4-quinolinyl)amino]benzoic acid (0.1 mmol) in 0.6 mL DMF was added the alcohol (0.3 mmol), a heaped microspatula of PyBOP, and DIPEA (0.052 mL, 0.3 mmol). The resultant solution was shaken for 10 minutes, and left overnight at room temperature. The sample was purified by Mass Directed Auto Prep (Sunfire C18 column, MeCN/H_2_O with formic acid modifier), and the solvent removed under a stream of nitrogen to afford 2-(dimethylamino)ethyl 2-((7-chloroquinolin-4-yl)amino)benzoate (15.4 mg, 33% yield).

**LCMS** (system A): t_ret_ 0.48 min, MH+ 370/372

**^1^H NMR** (400 MHz, CD_3_OD) δ ppm 8.56 (d, *J* = 5.5 Hz, 1H), 8.41 (br s, 1H), 8.24 (d, *J* = 9.1 Hz, 1H), 8.21 (dd, *J* = 8.1, 1.5 Hz, 1H), 7.95 (d, *J* = 2.0 Hz, 1H), 7.72-7.77 (m, 1H), 7.61-7.69 (m, 2H), 7.34 (d, *J* = 5.5 Hz, 1H), 7.18-7.24 (m, 1H), 4.59-4.67 (m, 2H), 3.35-3.40 (m, 2H), 2.81 ppm (s, 6H)

**Preparation of (2-((6,7-dimethoxyquinolin-4-yl)amino)phenyl)(4-methylpiperazin-1-yl)methanone, 23**

**

The aniline derivative (1 mmol) was dissolved in isopropanol (6 mL), and 1 mL dispensed into CEM microwave tubes. To each tube was added the heterocyclic chloride (0.16 mmol) and 2M HCl (0.048 mL). The tube was sealed, and heated in a CEM Discover microwave reactor using initial power of 150 W to 140 ^o^C for 10 minutes. The solvent was removed under a stream of nitrogen, redissolved in DMSO (0.5 mL), and purified by Mass Directed Auto Prep (Sunfire C18 column, MeCN/H_2_O with formic acid modifier), and the solvent removed under a stream of nitrogen to afford (2-((6,7-dimethoxyquinolin-4-yl)amino)phenyl)(4-methylpiperazin-1-yl)methanone (2.6 mg, 4% yield).

**LCMS** (system A): t_ret_ 0.47 min, MH+ 407

**^1^H NMR** (400 MHz, CDCl_3_) δ ppm 9.22-9.37 (m, 1H), 8.59 (s, 1H), 8.38 (d, *J* = 6.0 Hz, 1H), 7.65 (d, *J* = 8.1 Hz, 1H), 7.57 (s, 1H), 7.43-7.54 (m, 1H), 7.38 (dd, *J* = 7.6, 1.5 Hz, 1H), 7.30 (s, 1H), 7.19-7.24 (m, 1H), 7.00 (d, *J* = 6.0 Hz, 1H), 4.09 (s, 3H), 4.06 (s, 3H), 3.52-3.82 (m, 4H), 2.31-2.63 (m, 4H), 2.24 ppm (s, 3H)

**Preparation of 2-((7-chloroquinolin-4-yl)amino)-*N*-(2,3-dihydroxypropyl)-4,5-dimethylthiophene-3-carboxamide, 24**

1-(2,2-Dimethyl-1,3-dioxolan-4-yl)methanamine (0.016 g, 0.12 mmol) was weighed into tubes, followed by triazabicyclo[4.4.0]dec-5-ene (4.2 mg, 0.03 mmol). A solution of ester (0.1 mmol) in THF (0.6 mL) was dispensed into the tube, and the solution stirred at 75 ^o^C for 12 hours, and the solvent was removed under a stream of nitrogen. The sample was redissolved in DMSO (0.5 mL), and purified by Mass Directed Auto Prep (Sunfire C18 column, MeCN/H_2_O with formic acid modifier), and the solvent removed under a stream of nitrogen to afford 2-((7-chloroquinolin-4-yl)amino)-*N*-(2,3-dihydroxypropyl)benzamide (6.0 mg, 15% yield).

**LCMS** (system A): t_ret_ 0.59 min, MH+ 406/408

**^1^H NMR** (400 MHz, CD_3_OD) δ ppm 8.46-8.53 (m, 2H), 7.98 (d, *J* = 2.0 Hz, 1H), 7.80 (dd, *J* = 9.1, 2.0 Hz, 1H), 6.86 (d, *J* = 7.1 Hz, 1H), 3.47-3.56 (m, 1H), 3.35 (s, 2H), 3.17-3.26 (m, 2H), 2.42 (s, 3H), 2.23 ppm (s, 3H)

**Preparation of (tetrahydro-2*H*-pyran-4-yl)methyl 2-((7-chloroquinolin-4-yl)amino)benzoate, 25**

To a vial containing 2-[(7-chloro-4-quinolinyl)amino]benzoic acid (0.1 mmol) in 0. 6mL DMF was added the alcohol (0.3 mmol), a heaped microspatula of PyBOP, and DIPEA (0.052 mL, 0.3 mmol). The resultant solution was shaken for 10 minutes, and left overnight at room temperature. The sample was purified by Mass Directed Auto Prep (Sunfire C18 column, MeCN/H_2_O with formic acid modifier), and the solvent removed under a stream of nitrogen to afford (tetrahydro-2H-pyran-4-yl)methyl 2-((7-chloroquinolin-4-yl)amino)benzoate (10.3 mg, 23% yield).

**LCMS** (system A): t_ret_ 0.77 min, MH+ 397/399

**^1^H NMR** (400 MHz, CD_3_OD) δ ppm 8.53 (d, *J* = 6.0 Hz, 1H), 8.26 (d, *J* = 9.1 Hz, 1H), 8.13 (dd, *J* = 8.1, 1.5 Hz, 1H), 7.94 (d, *J* = 2.0 Hz, 1H), 7.61-7.75 (m, 3H), 7.21-7.28 (m, 2H), 4.18 (d, *J* = 6.0 Hz, 2H), 3.85-3.97 (m, 2H), 3.28-3.42 (m, 2H), 1.91-2.05 (m, 1H), 1.57-1.69 (m, 2H), 1.28-1.46 ppm (m, 2H)

**Preparation of (2-((7-chloroquinolin-4-yl)amino)phenyl)(1,1-dioxidothiomorpholino)methanone, 27**

HATU (4.8 mmol) was dissolved in 9.6 mL DMF, and added to 2-[(7-chloro-4-quinolinyl)amino]benzoic acid (720mg, 2.4mmol). To this solution was added DIPEA (1.25 mL, 7.2 mmol), and the resulting solution was shaking for 5 minutes. 0.452 mL of the solution (0.1 mmol acid, 0.2 mmol HATU, 0.3 mmol DIPEA) was added to the pre-weighed amine (0.1 mmol) in 0.2 mL DMF, the solution shaken for 10 minutes, and left overnight at room temperature. The sample was purified by Mass Directed AutoPrep (Sunfire C18 column, MeCN/H_2_O with formic acid modifier), and the solvent removed under a stream of nitrogen to afford (2-((7-chloroquinolin-4-yl)amino)phenyl)(1,1-dioxidothiomorpholino) methanone (10.5 mg, 23% yield).

**LCMS** (system A): t_ret_ 0.52 min, MH+ 416/418

**^1^H NMR** (400 MHz, DMSO-d_6_) δ ppm 8.32-8.40 (m, 2H), 8.15 (s, 1H), 7.88 (d, *J* = 2.0 Hz, 1H), 7.52-7.60 (m, 3H), 7.32-7.43 (m, 2H), 6.44 (d, *J* = 5.5 Hz, 1H), 2.97-3.22 ppm (m, 8H)

**Preparation of 6,7-bis(methyloxy)-*N*-[3-(1*H*-tetrazol-5-yl)phenyl]-4-quinolinamine, 28**

To a microwave vial containing methyl 3-(1*H*-tetrazol-5-yl) aniline (100 mg, 0.620 mmol) and 4-chloro-6,7-dimethoxyquinoline (139 mg, 0.620 mmol) was added isopropanol (4 mL), and 2 M HCl (0.31 mL), and the reaction heated to 140 ^o^C in a microwave reactor. The reaction mixture was diluted with 2M NaOH, and the aqueous phase washed with EtOAc (10 mL). The basic aqueous phase was acidified with 1M HCl, upon which product was observed to precipitate. The solid was filtered, washed with water, and dried *in vacuo* to afford 6,7-bis(methyloxy)-*N*-[3-(1*H*-tetrazol-5-yl)phenyl]-4-quinolinamine (15.0 mg, 7% yield).

**LCMS** (system A): t_ret_ 0.58 min, MH+ 349

**^1^H NMR** (400 MHz, DMSO-d_6_) δ ppm 10.02 (br s, 1H), 8.37 (d, *J* = 6.0 Hz, 1H), 8.12-8.16 (m, 1H), 8.02-8.05 (m, 1H), 7.94 (s, 2H), 7.58 (t, *J* = 8.1 Hz, 1H), 7.37-7.41 (m, 1H), 7.31-7.34 (m, 1H), 6.89 (d, *J* = 6.5 Hz, 1H), 3.99 (s, 3H), 3.97-3.98 (m, 1H), 3.97 ppm (s, 2H)

**Preparation of (2-((7-chloroquinolin-4-yl)amino)phenyl)(4-methylpiperazin-1-yl)methanone, 29**

The carboxylic acid (1.2 mmol) was dissolved in DMF (1.8 mL), added to a solution of HATU (1.2 mmol) and DIPEA (1.8 mmol) in DMF (1.8 mL), and the solution shaken for 10 minutes at room temperature. 0.65 mL of the solution was dispensed into a vial, amine (0.10 mmol) was added, the solution shaken for 10 minutes, and the reaction left overnight at room temperature. The reaction mixture was loaded onto a 1g aminopropyl SPE cartridge (pre-washed with 2 x 3 mL MeOH, and 6 mL chloroform), left for 1 hour to absorb, and eluted with 10% MeOH in EtOAC (2 x 3 mL), and the solvent removed *in vacuo*. The sample was dissolved in DMSO (0.5 mL) and purified by Mass Directed Auto Prep (Sunfire C18 column, MeCN/H_2_O with formic acid modifier), and the solvent removed under a stream of nitrogen to afford (2-((7-chloroquinolin-4-yl)amino)phenyl)(4-methylpiperazin-1-yl)methanone (15.8 mg, 37% yield).

**LCMS** (system A): t_ret_ 0.40 min, MH+ 381/383

**^1^H NMR** (400 MHz, CD_3_OD) δ ppm 8.35-8.42 (m, 2H), 8.27-8.33 (m, 1H), 7.94 (d, *J* = 2.0 Hz, 1H), 7.63-7.72 (m, 2H), 7.49-7.58 (m, 3H), 6.61 (d, *J* = 6.0 Hz, 1H), 3.39-3.67 (m, 4H), 3.28-3.34 (m, 4H), 2.33 ppm (s, 3H)

**Preparation of 2-((7-chloroquinolin-4-yl)amino)-*N*-(4-hydroxycyclohexyl) benzamide, 30**

The carboxylic acid (1.2 mmol) was dissolved in DMF (1.8 mL), added to a solution of HATU (1.2 mmol) and DIPEA (1.8 mmol) in DMF (1.8 mL), and the solution shaken for 10 minutes at room temperature. 0.65 mL of the solution was dispensed into a vial, amine (0.10 mmol) was added, the solution shaken for 10 minutes, and the reaction left overnight at room temperature. The reaction mixture was loaded onto a 1g aminopropyl SPE cartridge (pre-washed with 2 x 3 mL MeOH, and 6 mL chloroform), left for 1 hour to absorb, and eluted with 10% MeOH in EtOAC (2 x 3 mL), and the solvent removed *in vacuo*. The sample was dissolved in DMSO (0.5 mL) and purified by Mass Directed Auto Prep (Sunfire C18 column, MeCN/H_2_O with formic acid modifier), and the solvent removed under a stream of nitrogen to afford 2-((7-chloroquinolin-4-yl)amino)-*N*-(4-hydroxycyclohexyl)benzamide (11.2 mg, 25.5% yield).

**LCMS** (system A): t_ret_ 0.57 min, MH+ 396/398

**^1^H NMR** (400 MHz, DMSO-d_6_) δ ppm 10.61-10.78 (m, 1H), 8.51-8.61 (m, 1H), 8.38-8.50 (m, 1H), 8.08-8.21 (m, 1H), 7.88-8.00 (m, 1H), 7.73-7.79 (m, 1H), 7.59-7.71 (m, 2H), 7.46-7.57 (m, 1H), 7.14-7.23 (m, 1H), 7.05-7.12 (m, 1H), 4.43-4.68 (m, 1H), 3.58-3.72 (m, 1H), 3.25-3.47 (m, 1H), 1.64-1.86 (m, 4H), 1.06-1.40 ppm (m, 5H)

**Preparation of 4-(aminopiperidin-1-yl)(2-((5-chloropyrazolo[1,5-a]pyrimidin-7-yl)amino)phenyl) methanone, 31**

The carboxylic acid (0.176 mmol) was dissolved in DMF (0.8 mL), and added to a solution of HATU (0.35 mmol) in DMF. DIPEA (0.524 mmol) was added, and the solution shaken for 10 minutes at room temperature. 0.33 mL of the solution was dispensed into a vial, and amine (0.44 mmol) was added, the solution shaken for 10 minutes, and the reaction left overnight at room temperature. The reaction mixture was loaded onto a 1g aminopropyl SPE cartridge (pre-washed with 2 x 3 mL MeOH, and 6 mL chloroform), left for 1 hour to absorb, and eluted with 10% MeOH in EtOAC (2 x 3 mL), and the solvent removed *in vacuo*. The sample was dissolved in DMSO (0.5 mL) and purified by Mass Directed Auto Prep (Sunfire C18 column, MeCN/H_2_O with formic acid modifier), and the solvent removed under a stream of nitrogen to afford (4-aminopiperidin-1-yl)(2-((5-chloropyrazolo[1,5-a]pyrimidin-7-yl)amino)phenyl)methanone (4.8 mg, 27% yield).

**LCMS** (system A): t_ret_ 0.59 min, MH+ 371/373

**^1^H NMR** (400 MHz, DMSO-d_6_) δ ppm 8.29-8.38 (m, 1H), 8.13-8.22 (m, 1H), 7.53-7.60 (m, 2H), 7.36-7.46 (m, 2H), 6.49 (d, *J* = 2.5 Hz, 1H), 5.88-5.96 (m, 1H), 4.21-4.33 (m, 1H), 3.50-3.60 (m, 1H), 3.00-3.13 (m, 2H), 2.71-2.85 (m, 1H), 1.68-1.87 (m, 2H), 1.13-1.47 ppm (m, 2H)

**Preparation of *N*-(4-hydroxycyclohexyl)-2-(quinolin-4-ylamino)benzamide, 32**

The carboxylic acid (0.72 mmol) was dissolved in DMF (3 mL), added to a solution of HATU (1.44 mmol) and DIPEA (2.16 mmol) in DMF (1.8 mL), and the solution shaken for 10 minutes at room temperature. 0.86 mL of the solution was dispensed into a vial, amine (0.12 mmol) was added, the solution shaken for 10 minutes, and the reaction left overnight at room temperature. The reaction mixture was loaded onto a 1 g aminopropyl SPE cartridge (pre-washed with 2 x 3 mL MeOH, and 6 mL chloroform), left for 1 hour to absorb, and eluted with 10% MeOH in EtOAC (2 x 3 mL), and the solvent removed *in vacuo*. The sample was dissolved in DMSO (0.5 mL) and purified by Mass Directed Auto Prep (Sunfire C18 column, MeCN/H_2_O with formic acid modifier), and the solvent removed under a stream of nitrogen to afford *N*-(4-hydroxycyclohexyl)-2-(quinolin-4-ylamino)benzamide (15.4 mg, 32% yield).

**LCMS** (system A): t_ret_ 0.52 min, MH+ 362

**^1^H** **NMR** (400 MHz, DMSO-d_6_) δ ppm 8.46-8.57 (m, 2H), 8.08-8.15 (m, 1H), 7.87-7.94 (m, 1H), 7.71-7.80 (m, 2H), 7.58-7.67 (m, 2H), 7.53 (d, *J* = 7.1 Hz, 1H), 7.07-7.17 (m, 2H), 3.62-3.75 (m, 1H), 3.27-3.39 (m, 1H), 1.69-1.82 (m, 4H), 1.22 ppm (d, *J* = 10.1 Hz, 4H)

**Preparation of methyl 2-[(7-chloro-4-quinolinyl)(methyl)amino]benzoate, 33**

To a vial containing 2-[(7-chloro-4-quinolinyl)methylamino]benzoic acid (30 mg, 0.096 mmol) in 3 ml DMF was added (2,2-dimethyl-1,3-dioxolan-4-yl)methanol (0.3mmol), a heaped microspatula of PyBOP, and DIPEA (0.052 mL, 0.3 mmol). The resultant solution was shaken for 10 minutes, and left overnight at room temperature. No desired product was observed by LCMS, so MeOH (5 mL), further DIPEA (0.052 mL) and PyBOP were added, and the reaction stirred for 3 hours at room temperature. The solvent was removed under a stream of nitrogen, and the crude residue purified by flash column chromatography (SiO_2_) eluting with 0-25% MeOH in DCM. The solvent was removed *in vacuo* to afford (tetrahydro-2H-pyran-4-yl)methyl methyl 2-[(7-chloro-4-quinolinyl)(methyl)amino]benzoate (6.0 mg, 19% yield).

**LCMS** (system A): t_ret_ 0.72 min, MH+ 327/329

**^1^H NMR** (400 MHz, CD_3_OD) δ ppm 8.60 (br d, *J* = 6.0 Hz, 1H), 7.83-7.91 (m, 2H), 7.61-7.68 (m, 1H), 7.43-7.49 (m, 1H), 7.26-7.38 (m, 2H), 7.06-7.17 (m, 2H), 3.60 (s, 3H), 3.56 ppm (s, 3H)

**Preparation of (tetrahydro-2*H*-pyran-2-yl)methyl 2-((7-chloroquinolin-4-yl)amino)benzoate, 34**

To a vial containing 2-[(7-chloro-4-quinolinyl)amino]benzoic acid (0.1 mmol) in 0.5 ml DMF was added the alcohol (0.3 mmol), a heaped microspatula of PyBOP, and DIPEA (0.052 mL, 0.3 mmol). The resultant solution was shaken for 10 minutes, and left overnight at room temperature. The sample was purified by Mass Directed Auto Prep (Sunfire C18 column, MeCN/H_2_O with formic acid modifier), and the solvent removed under a stream of nitrogen to afford (tetrahydro-2*H*-pyran-2-yl)methyl 2-((7-chloroquinolin-4-yl)amino)benzoate (10.3 mg, 23% yield).

**LCMS** (system A): t_ret_ 0.87 min, MH+ 397/399

**^1^H NMR** (400 MHz, CD_3_OD) δ ppm 8.54 (d, *J* = 5.5 Hz, 1H), 8.20 (d, *J* = 9.1 Hz, 1H), 8.13 (dd, *J* = 7.6, 1.5 Hz, 1H), 7.92 (d, *J* = 2.0 Hz, 1H), 7.68-7.74 (m, 1H), 7.56-7.66 (m, 2H), 7.32 (d, *J* = 5.5 Hz, 1H), 7.12-7.21 (m, 1H), 4.24-4.29 (m, 2H), 3.84-3.93 (m, 1H), 3.61-3.69 (m, 1H), 3.37-3.45 (m, 1H), 1.81-1.91 (m, 1H), 1.59-1.67 (m, 1H), 1.47-1.58 (m, 3H), 1.31-1.45 ppm (m, 1H)

**Preparation of methyl 2-[(2-methyl-4-quinolinyl)amino]benzoate, 35**

To a microwave vial containing methyl 2-aminobenzoate (0.073 mL, 0.563 mmol) and 4-chloro-2-methylquinoline (0.114 mL, 0.563 mmol) was added isopropanol (4 mL), and HCl (1 mL, 32.9 mmol), and the reaction heated to 140 ^o^C in a microwave reactor for 1 hour. The solvent was removed *in vacuo*, and the crude residue purified by flash column chromatography (SiO_2_, 70g) eluting with 0-25% MeOH in DCM. The solvent was removed *in vacuo*, and the residue further purified by Mass Directed Auto Prep (Sunfire C18 column, MeCN/H_2_O with formic acid modifier), and the solvent removed under a stream of nitrogen to afford methyl 2-[(2-methyl-4-quinolinyl)amino]benzoate (8 mg, 5% yield).

**LCMS** (system A): t_ret_ 0.70 min, MH+ 293

**^1^H NMR** (400MHz, CD_3_OD) δ ppm 8.43-8.46 (m, 2H), 8.20 (dd, *J* = 7.8 Hz, 1H), 7.92-8.00 (m, 2H), 7.74-7.81 (m, 3H) 7.48 (td, *J* = 7.4, 1.8 Hz, 1H), 6.94 (s, 1H), 3.89 (s, 3H), 2.66 (s, 3H)

**Preparation of (4-((5-chloropyrazolo[1,5-a]pyrimidin-7-yl)amino)phenyl)(4-methylpiperazin-1-yl)methanone, 36**

4-((5-Chloropyrazolo[1,5-a]pyrimidin-7-yl)amino)benzoic acid (29 mg, 0.10 mmol) and HATU (38 mg, 0.1 mmol) were dissolved in DMF (0.4 mL), DIPEA (0.050 mL, 0.29 mmol) was added to the solution, which was sealed, and sonicated for 1 minute to aid dispersion 1-Methylpiperazine was added, and the vial shaken, then left to stand at room temperature for 48 hours. The solution was diluted with DMSO (0.2 mL), purified by Mass Directed Auto Prep (Sunfire C18 column, MeCN/H_2_O with formic acid modifier), and the solvent removed under a stream of nitrogen to afford (4-aminopiperidin-1-yl)(2-((5-chloropyrazolo[1,5-a]pyrimidin-7-yl)amino)phenyl)methanone (12.7 mg, 27% yield).

**LCMS** (system A): t_ret_ 0.56 min, MH+ 371/373

**^1^H NMR** (600 MHz, DMSO-d_6_) δ ppm 8.20-8.29 (m, 1H), 7.52-7.59 (m, 2H), 7.42-7.51 (m, 2H), 6.51-6.60 (m, 1H), 6.20-6.35 (m, 1H), 5.71-5.79 (m, 1H), 3.30-3.84 (m, 4H), 2.26-2.43 (m, 4H), 2.12-2.24 ppm (m, 3H)

**Preparation of 2-ethoxyethyl 2-((7-chloroquinolin-4-yl)amino)benzoate, 38**

To a vial containing 2-[(7-chloro-4-quinolinyl)amino]benzoic acid (0.1 mmol) in 0.6 ml DMF was added the alcohol (0.3 mmol), a heaped microspatula of PyBOP, and DIPEA (0.052 mL, 0.3 mmol). The resultant solution was shaken for 10 minutes, and left overnight at room temperature. The sample was purified by Mass Directed Auto Prep (Sunfire C18 column, MeCN/H_2_O with formic acid modifier), and the solvent removed under a stream of nitrogen to afford 2-ethoxyethyl 2-((7-chloroquinolin-4-yl)amino)benzoate (8.4 mg, 20% yield).

**LCMS** (system A): t_ret_ 0.76 min, MH+ 371/373

**^1^H NMR** (400MHz, CD_3_OD) δ ppm 8.57 (d, *J* = 5.5 Hz, 1H), 8.23 (d, *J* = 9.1 Hz, 1H), 8.16 (dd, *J* = 8.1 Hz, 1.5 Hz, 1H), 7.95 (d, *J* = 2.5 Hz, 1H), 7.75 (d, *J* = 8.1 Hz, 1H), 7.61-7.68 (m, 2H), 7.38 (d, *J* = 5.5 Hz, 1H), 7.20 (d, *J* = 8.6 Hz, 1H), 4.46-4.51 (m, 2H), 3.74-3.79 (m, 2H), 3.55 (q, *J* = 7.05 Hz, 2H), 1.16 (t, *J* =6.8 Hz, 3H)

**Preparation of 2-aminoethyl 2-((7-chloroquinolin-4-yl)amino)benzoate, 39**

To a vial containing 2-[(7-chloro-4-quinolinyl)amino]benzoic acid (0.1 mmol) in 0.6 ml DMF was added the alcohol (0.3 mmol), a heaped microspatula of PyBOP, and DIPEA (0.052 mL, 0.3 mmol). The resultant solution was shaken for 10 minutes, and left overnight at room temperature. The sample was purified by Mass Directed Auto Prep (Sunfire C18 column, MeCN/H_2_O with formic acid modifier), and the solvent removed under a stream of nitrogen to afford 2-aminoethyl 2-((7-chloroquinolin-4-yl)amino)benzoate (18 mg, 47% yield).

**LCMS** (system A): t_ret_ 0.48 min, MH+ 342/344

**^1^H NMR** (400MHz, DMSO-d_6_) δ ppm 8.60 (d, *J* = 9.1 Hz, 1H), 8.45 (d, *J* =7.05 Hz, 1H), 8.40 (dd, *J* =7.55, 1.5 Hz, 1H), 8.02 (d, *J* = 2.0 Hz, 1H), 7.86-7.91 (m, 2H), 7.76 (dd, *J* = 8.1, 1.0 Hz, 1H), 7.64 (d, *J* = 8.1 Hz, 1H), 6.91 (d, *J* 7.05 Hz, 1H), 4.51-4.54 (m, 2H), 3.35-3.38 (m, 2H), 2.68 (br s, 2H).

**Preparation of (4-((6,7-dimethoxyquinolin-4-yl)amino)phenyl)(pyrrolidin-1-yl)methanone, 40**

4-{[6,7-bis(Methoxy)-4-quinolinyl]amino}benzoic acid (50 mg, 0.12 mmol) and HATU (50 mg, 0.13 mmol) were dissolved in DMF (0.4 mL), and stirred for 10 minutes. Pyrrolidine (0.12 mmol) and DIPEA (0.063 mL, 0.0.36 mmol) were added to the solution, and the reaction stirred for 72 hours. The solution was diluted with 1:1 MeOH/DMSO (0.2 mL), purified by Mass Directed Auto Prep (Sunfire C18 column, MeCN/H_2_O with formic acid modifier), and the solvent removed under a stream of nitrogen to afford (4-((6,7-dimethoxyquinolin-4-yl)amino)phenyl)(pyrrolidin-1-yl)methanone (9.3 mg, 19% yield).

**LCMS** (system A): t_ret_ 0.65 min, MH+ 378

**^1^H NMR** (600 MHz, DMSO-d_6_) δ ppm 8.89 (br s, 1H), 8.35 (d, *J* = 4.9 Hz, 1H), 8.21 (s, 1H), 7.63 (s, 1H), 7.57 (d, *J* = 8.7 Hz, 1H), 7.34 (d, *J* = 8.7 Hz, 1H), 7.28 (s, 1H), 7.01 (d, *J* = 4.9 Hz, 1H), 3.93 (s, 3H), 3.91 (s, 3H), 3.39-3.72 (m, 2H), 1.76-1.93 ppm (m, 2H)

**Preparation of methyl 2-{[2-(trifluoromethyl)-4-quinolinyl]amino}benzoate, 41**

A solution of Pd(OAc)_2_ (5.9 mg, 0.025 mmol) and BINAP (33 mg, 0.053 mmol) in toluene (5 mL) were stirred under air in a microwave vial for 15 minutes, after which methyl 2-aminobenzoate (0.171 mL, 1.32 mmol), 4-bromo-2-(trifluoromethyl)quinoline (349 mg, 1.26 mmol) and potassium phosphate (281 mg, 1.32 mmol) were added. The vial was sealed and heated in a Biotage Initiator microwave reactor for 1 hour at 80 ^o^C. The reaction mixture was filtered, and the diluted with brine and EtOAc. The organic phase was separated, dried through a hydrophobic filter, and the solvent removed *in vacuo*. The crude residue was purified by flash column chromatography (SiO2) eluting with 0-100% EtOAc in cyclohexane, and the solvent removed *in vacuo*. The sample was further purified by Mass Directed Auto Prep (Sunfire C18 column, MeCN/H_2_O with formic acid modifier), and the solvent removed under a stream of nitrogen to afford methyl 2-{[2-(trifluoromethyl)-4-quinolinyl]amino}benzoate (24.4 mg, 5% yield).

**LCMS** (system A): t_ret_ 1.36 min, MH+ 347

**^1^H NMR** (400 MHz, CD_3_OD) δ ppm 8.28-8.34 (m, 1H), 8.16 (dd, *J* = 7.8, 1.8 Hz, 1H), 8.08-8.13 (m, 1H), 7.85-7.93 (m, 1H), 7.74-7.80 (m, 2H), 7.65-7.72 (m, 1H), 7.53-7.58 (m, 1H), 7.20-7.28 (m, 1H), 3.93 ppm (s, 3H)

**Preparation of (4-methylpiperazin-1-yl)(2-(thieno[3,2-b]pyridin-7-ylamino) phenyl)methanone, 43**

The aniline derivative (1 mmol) was dissolved in isopropanol (6 mL), and 1 mL dispensed into CEM microwave tubes. To each tube was added the heterocyclic core (0.16 mmol) and 2M HCl (0.048 mL). The tube was sealed, and heated in a CEM Discover microwave reactor using initial power of 150 W to 140 ^o^C for 20 minutes. The solvent was removed under a stream of nitrogen, redissolved in DMSO (0.5 mL), and purified by Mass Directed Auto Prep (Sunfire C18 column, MeCN/H_2_O with formic acid modifier), and the solvent removed under a stream of nitrogen to afford (4-methylpiperazin-1-yl)(2-(thieno[3,2-b]pyridin-7-ylamino)phenyl)methanone (3.2 mg, 5% yield).

**LCMS** (system A): t_ret_ 0.37 min, MH+ 353

**^1^H NMR** (400 MHz, CD_3_OD) δ ppm 8.23 (d, *J* = 5.5 Hz, 1H), 7.89 (d, *J* = 5.5 Hz, 1H), 7.53-7.60 (m, 1H), 7.36-7.47 (m, 4H), 6.67 (d, *J* = 5.5 Hz, 1H), 3.46-3.61 (m, *J* = 2.0 Hz, 2H), 2.16-2.27 (m, 2H), 2.13 ppm (s, 3H)

**Preparation of methyl 2-((7-chloro-2-methylquinolin-4-yl)amino)benzoate, 44**

Methyl 2-aminobenzoate (71 mg, 0.47 mmol) and 4,7-dichloro-2-methylquinoline (100 mg, 0.47 mmol) were dissolved in isopropanol (4 mL) and 1M HCl (1 mL). The tube was sealed, and heated in a CEM Discover microwave reactor using initial power of 150 W to 140 ^o^C for 1 hour. The solvent was removed under a stream of nitrogen, and the crude material purified by flash column chromatography (SiO_2_) eluting with 0-25% MeOH in DCM, and the solvent removed *in vacuo* to afford methyl 2-((7-chloro-2-methylquinolin-4-yl)amino)benzoate (4.5 mg, 3% yield).

**LCMS** (system A): t_ret_ 0.76 min, MH+ 327/329

**^1^H-NMR** (400 MHz, CDCl_3_)  ppm 10.48 (br s, 1H), 8.11 (dd, *J* = 8.1 Hz, 1.5 Hz, 1H), 7.96-8.08 (m, 2H), 7.67 (d, *J* = 8.1 Hz, 1 H), 7.51-7.61 (m, 1H), 7.48 (dd, *J* = 8.8, 2.0 Hz, 1H), 7.33 (s, 1H), 7.04 (t, *J*= 7.55 Hz, 1H), 3.98 (s, 3H), 2.69 (s, 3H)

**Preparation of 2-(pyrido[3,2-d]pyrimidin-4-ylamino)benzoic acid, 45**

2-Aminobenzoic acid (83 mg, 0.60 mmol) and 4-chloropyrido[3,2-d]pyrimidine (100 mg, 0.60 mmol) were dissolved in isopropanol (4 mL). The tube was sealed, and heated in a microwave reactor at 140 ^o^C for 1 hour. The solvent was removed under a stream of nitrogen, and the crude material purified by Mass Directed Auto Prep (Sunfire C18 column, MeCN/H_2_O with formic acid modifier), and the solvent removed under a stream of nitrogen to afford 2-(pyrido[3,2-d]pyrimidin-4-ylamino)benzoic acid (130 mg, 81% yield).

**LCMS** (system A): t_ret_ 0.69 min, MH+ 267

**^1^H NMR** (400MHz, DMSO-d_6_)  ppm 12.80 (br s, 1H), 9.27 (dd, *J* = 8.6, 1.0 Hz, 1H), 8.99 (dd, *J* = 4.0, 1.5 Hz, 1H), 8.83 (s, 1H), 8.29 (dd, *J* = 8.5, 1.5 Hz, 1H), 8.10 (dd, *J* = 8.0, 1.5 Hz, 1H), 7.96 (dd, *J* =8.5, 4.0 Hz, 1H), 7.73 (ddd, *J* = 8.5, 7.0, 1.5 Hz, 1H), 7.22 (d, *J* = 7.0 Hz, 1H).

**Preparation of 2-((7-chloroquinolin-4-yl)amino)-*N*-(tetrahydro-2*H*-pyran-4-yl)benzamide, 46**

HATU (4.8 mmol) was dissolved in 9.6 mL DMF, and added to 2-[(7-chloro-4-quinolinyl)amino]benzoic acid (720mg, 2.4mmol). To this solution was added DIPEA (1.25 mL, 7.2 mmol), and the resulting solution was shaking for 5 minutes. 0.452 mL of the solution was added to the pre-weighed amine (0.1 mmol) in 0.2 mL DMF, the solution shaken for 10 minutes, and left overnight at room temperature. The sample was purified by Mass Directed AutoPrep (Sunfire C18 column, MeCN/H_2_O with formic acid modifier), and the solvent removed under a stream of nitrogen to afford 2-((7-chloroquinolin-4-yl)amino)-*N*-(tetrahydro-2*H*-pyran-4-yl)benzamide (8.5 mg, 20% yield).

**LCMS** (system A): t_ret_ 0.61 min, MH+ 382/384

**^1^H NMR** (400MHz, DMSO-d_6_) δ ppm 8.63 (d, *J* = 7.55 Hz, 1H), 8.51 (d, *J* = 5.0 Hz, 1H), 8.14-8.28 (m, 2H), 7.93 (d, *J* = 2.0 Hz, 1H), 7.79 (dd, *J* = 7.8, 1.3 Hz, 1H), 7.60-7.67 (m, 1H), 7.52-7.57 (m, 1H), 7.11-7.25 (m, 1H), 7.06 (d, *J* = 5.0 Hz, 1H), 3.73-3.84 (m, 2H), 3.28-3.34 (m, 4H), 1.63 (dd, *J* = 12.3, 2.3 Hz, 2H), 1.40-1.50 (m, 2H)

**Preparation of 4-(thieno[3,2-b]pyridin-7-ylamino)benzoic acid, 47**

4-Aminobenzoic acid (323 mg, 2.36 mmol) and 7-chlorothieno[3,2-b]pyridine (400 mg, 2.36 mmol) were dissolved in isopropanol (4 mL) and 2M HCl (1.2 mL). The tube was sealed, and heated in a microwave reactor at 140 ^o^C for 1 hour. The reaction mixture was basified with excess 2M NaOH, and washed with EtOAc. The basic aqueous phase was acidified with 2M HCl, upon which product was observed to precipitate. The solid was filtered, washed with water, and dried *in vacuo* to afford 4-(thieno[3,2-b]pyridin-7-ylamino)benzoic acid (0.634 g, 88% yield).

**LCMS** (system B): t_ret_ 1.10 min, MH+ 271

**^1^H NMR** (400 MHz, DMSO-d_6_) δ ppm 12.75 (br s, 1H), 9.93 (br s, 1H), 8.43 (d, *J* = 5.5 Hz, 1H), 8.16 (d, *J* = 5.5 Hz, 1H), 7.86-8.02 (m, 2H), 7.55 (d, *J* = 5.5 Hz, 1H), 7.32-7.46 (m, 2H), 7.14 ppm (d, *J* = 6.0 Hz, 1H)

**Preparation of 2-ethenyl-3-(methylthio)-*N*-[2-(1*H*-tetrazol-5-yl)phenyl]-4-pyridinamine, 48**

2-(1*H*-Tetrazol-5-yl)aniline (100 mg, 0.62 mmol) and 7-chlorothieno[3,2-b]pyridine (105 mg, 0.62 mmol) were dissolved in isopropanol (4 mL) and 2M HCl (1 mL). The tube was sealed, and heated in a microwave reactor at 140 ^o^C for 1 hour. The solvent was removed *in vacuo*, and the crude residue purified by flash column chromatography (SiO_2_, 100g) eluting with 0-50% MeOH in DCM. The sample was further purified by Mass Directed AutoPrep (Sunfire C18 column, MeCN/H_2_O with formic acid modifier), and the solvent removed under a stream of nitrogen to afford 2-ethenyl-3-(methylthio)-*N*-[2-(1*H*-tetrazol-5-yl)phenyl]-4-pyridinamine (10 mg, 5.5% yield).

**LCMS** (system A): t_ret_ 0.50 min, MH+ 295

**^1^H NMR** (400 MHz, CD_3_OD) δ ppm 8.25 (d, *J* = 6.5 Hz, 1H), 8.10-8.20 (m, 2H), 7.65-7.72 (m, 1H), 7.53 (td, *J* = 7.6, 1.5 Hz, 1H), 7.39-7.50 (m, 2H), 7.06-7.13 ppm (m, 1H)

**Preparation of 3-(thieno[3,2-b]pyridin-7-ylamino)benzoic acid, 49**

3-Aminobenzoic acid (323 mg, 2.36 mmol) and 7-chlorothieno[3,2-b]pyridine (400 mg, 2.36 mmol) were dissolved in isopropanol (4 mL) and 2M HCl (1.2 mL). The tube was sealed, and heated in a microwave reactor at 140 ^o^C for 1 hour. The reaction mixture was basified with excess 2M NaOH, and washed with EtOAc. The basic aqueous phase was acidified with 2M HCl, upon which product was observed to precipitate. The solid was filtered, washed with water, and dried *in vacuo* to afford 3-(thieno[3,2-b]pyridin-7-ylamino)benzoic acid (0.521 g, 72% yield).

**LCMS** (system B): t_ret_ 1.07 min, MH+ 271

**^1^H-NMR** (400 MHz, DMSO-d6)  ppm 6.95 (d, *J*=5.54 Hz, 1 H), 7.44 - 7.56 (m, 3 H), 7.63 - 7.69 (m, 1 H), 7.84 (s, 1 H), 8.02 (d, *J*=5.29 Hz, 1 H), 8.36 (d, *J*=5.29 Hz, 1 H), 9.06 (s, 1 H), 13.05 (br s, 1 H)

**Preparation of (2-((5-chloropyrazolo[1,5-a]pyrimidin-7-yl)amino)phenyl)(4-hydroxypiperidin-1-yl)methanone, 50**

2-((5-Chloropyrazolo[1,5-a]pyrimidin-7-yl)amino)benzoic acid (29 mg, 0.10 mmol) and HATU (38 mg, 0.1 mmol) were dissolved in DMF (0.4 mL), DIPEA (0.050 mL, 0.29 mmol) was added to the solution, which was sealed, and sonicated for 1 minute to aid dispersion. 4-Hydroxypiperidine (0.090 mmol) was added, and the vial shaken and then left to stand at room temperature for 18 hours. Further HATU (29 mg) in 0.2 mL DMF, and DIPEA (0.025 mL) were added, and the reaction left to stand at room temperature for a further 18 hours. The solution was diluted with DMSO (0.2 mL), purified by Mass Directed Auto Prep (Sunfire C18 column, MeCN/H_2_O with formic acid modifier), and the solvent removed under a stream of nitrogen to afford (2-((5-chloropyrazolo[1,5-a]pyrimidin-7-yl)amino)phenyl)(4-hydroxypiperidin-1-yl)methanone (14.0 mg, 41% yield).

**LCMS** (system A): t_ret_ 0.80 min, MH+ 372/374

**^1^H NMR** (600 MHz, DMSO-d_6_) δ ppm 8.15-8.26 (m, 1H), 7.57 (br s, 2H), 7.38-7.50 (m, 2H), 6.51 (s, 1H), 5.87-5.97 (m, 1H), 5.65-5.80 (m, 1H), 3.80-3.91 (m, 1H), 3.63-3.70 (m, 1H), 3.26-3.53 (m, 3H), 3.04-3.15 (m, 2H), 1.59-1.68 (m, 2H), 1.20-1.34 ppm (m, 2H)

**Preparation of 2-(thieno[3,2-*b*]pyridin-7-ylamino)benzoic acid, 51**

2-Aminobenzoic acid (323 mg, 2.36 mmol) and 7-chlorothieno[3,2-b]pyridine (400 mg, 2.36 mmol) were dissolved in isopropanol (4 mL) and 2M HCl (1.2 mL). The tube was sealed, and heated in a microwave reactor at 140 ^o^C for 1 hour. The reaction mixture was basified with excess 2M NaOH, and washed with EtOAc. The basic aqueous phase was acidified with 2M HCl, upon which product was observed to precipitate. The solid was filtered, washed with water, and dried *in vacuo* to afford 2‑(thieno[3,2-*b*]pyridin-7-ylamino)benzoic acid (0.531 g, 73.5% yield).

**LCMS** (system B): t_ret_ 1.25 min, MH+ 271

**^1^H NMR** (400 MHz, DMSO-d_6_) δ ppm 13.23 (br s, 1H), 8.37 (d, *J* = 5.5 Hz, 1H), 7.98-8.05 (m, 2H), 7.49-7.53 (m, 1H), 7.47 (d, *J* = 5.5 Hz, 1H), 7.31-7.36 (m, 1H), 7.26 (d, *J* = 5.5 Hz, 1H), 6.89-6.95 ppm (m, 1H)

**Preparation of (2-((5-chloropyrazolo[1,5-a]pyrimidin-7-yl)amino)phenyl)(morpholino)methanone, 52**

2-((5-Chloropyrazolo[1,5-a]pyrimidin-7-yl)amino)benzoic acid (29 mg, 0.10 mmol) and HATU (38 mg, 0.1 mmol) were dissolved in DMF (0.4 mL), DIPEA (0.050 mL, 0.29 mmol) was added to the solution, which was sealed, and sonicated for 1 minute to aid dispersion. Morpholine (0.090 mmol) was added, and the vial shaken, and then left to stand at room temperature for 18 hours. Further HATU (29 mg) in 0.2 mL DMF, and DIPEA (0.025 mL) were added, and the reaction left to stand at room temperature for a further 18 hours. The solution was diluted with DMSO (0.2 mL), purified by Mass Directed Auto Prep (Atlantis column, MeCN/H_2_O with formic acid modifier), and the solvent removed under a stream of nitrogen to afford (2-((5-chloropyrazolo[1,5-a]pyrimidin-7-yl)amino)phenyl)(morpholino) methanone (16.4 mg, 50% yield).

**LCMS** (system A): t_ret_ 0.89 min, MH+ 358/360

**^1^H NMR** (600 MHz, DMSO-d_6_) δ ppm 10.23 (br s, 1H), 8.19-8.27 (m, 1H), 7.40-7.63 (m, 4H), 6.47-6.58 (m, 1H), 5.80-5.91 (m, 1H), 3.16-3.77 ppm (m, 8H)

**Preparation of 2-((7-chloroquinolin-4-yl)amino)benzoic acid, 53**

5 M aq. NaOH (0.19 mL, 0.956 mmol) was added to a solution of methyl 2-((7-chloroquinolin-4-yl)amino)benzoate (500 mg) in MeOH (12 mL) and the reaction heated in a microwave reactor at 150 ^o^C for 15 minutes. The sample was acidified to pH1 with 2M HCl and concentrated *in vacuo*. The crude residue was dissolved in MeOH and filtered twice to remove insoluble impurities, and the solvent removed *in vacuo* to afford 2-((7-chloro-quinolin-4-yl)amino)benzoic acid (209 mg, 73% yield).

**LCMS** (system A): t_ret_ 0.58 min, MH+ 299/301

**^1^H NMR** (400MHz, DMSO-d6) δ ppm 11.15 (br s, 1H), 8.74 (d, *J* = 9.1 Hz, 1H), 8.52 (d, *J* = 7.1 Hz, 1H), 8.18 (br s, 1H), 8.10 (d, *J* = 8.6 Hz, 1H), 7.94 (dd, *J* 9.1, 1.5 Hz, 1H), 7.64-7.78 (m, 1H), 7.57-7.66 (m, 2H), 6.58 (d, *J* = 7.1 Hz, 1H)

**Preparation of 2-((7-chloroquinolin-4-yl)amino)-*N*-(tetrahydro-2*H*-pyran-3-yl)benzamide, 54**

HATU (4.8 mmol) was dissolved in 9.6 mL DMF, and added to 2-[(7-chloro-4-quinolinyl)amino]benzoic acid (720mg, 2.4mmol). To this solution was added DIPEA (1.25 mL, 7.2 mmol), and the resulting solution was shaking for 5 minutes. 0.452 mL of the solution was added to the pre-weighed amine (0.1 mmol) in 0.2 mL DMF, the solution shaken for 10 minutes, and left overnight at room temperature. The sample was purified by Mass Directed AutoPrep (Sunfire C18 column, MeCN/H_2_O with formic acid modifier), and the solvent removed under a stream of nitrogen to afford 2-((7-chloroquinolin-4-yl)amino)-*N*-(tetrahydro-2*H*-pyran-3-yl)benzamide (11.1 mg, 26% yield)

**LCMS** (system A): t_ret_ 0.63, MH+ 382/384

**^1^H NMR** (400 MHz, DMSO-d_6_) δ ppm 8.53 (br d, *J* = 7.1 Hz, 1H), 8.46 (br d, *J* = 5.0 Hz, 1H), 8.15-8.22 (m, 2H), 7.91 (d, *J* = 2.0 Hz, 1H), 7.76 (d, *J* = 7.1 Hz, 1H), 7.63 (dd, *J* = 9.1, 2.5 Hz, 1H), 7.50-7.59 (m, 2H), 7.15-7.24 (m, 1H), 6.95 (br d, *J* = 5.0 Hz, 1H), 3.73-3.84 (m, 1H), 3.15-3.25 (m, 2H), 2.97-3.08 (m, 2H), 1.72-1.82 (m, 1H), 1.53-1.64 (m, 1H), 1.41-1.49 ppm (m, 2H)

**Preparation of *N*-(3-(1*H*-tetrazol-5-yl)phenyl)-5-chloropyrazolo[1,5-*a*]pyrimidin-7-amine, 55**

To a microwave vial containing methyl 3-(1H-tetrazol-5-yl)aniline (100 mg, 0.620 mmol) and 5,7-dichloropyrazolo[1,5-a]pyrimidine (117 mg, 0.620 mmol) was added isopropanol (4 mL), and 2 M HCl (0.31 mL), and the reaction heated to 140 ^o^C in a microwave reactor. The reaction mixture was diluted with 2M NaOH, and the aqueous phase washed with EtOAc (10 mL). The basic aqueous phase was acidified with 1M HCl, upon which product was observed to precipitate. The solid was filtered, washed with water, and dried *in vacuo*. The solid was further purified by Mass Directed AutoPrep (Sunfire C18 column, MeCN/H_2_O with formic acid modifier) to afford *N*-(3-(1*H*-tetrazol-5-yl)phenyl)-5-chloropyrazolo[1,5-*a*]pyrimidin-7-amine (41.0 mg, 21% yield).

**LCMS** (system B): t_ret_ 2.20 min, MH+ 313/315

**^1^H NMR** (400MHz, DMSO-d_6_) δ ppm 10.60 (br s, 1H), 8.28 (d, *J* = 2.0 Hz, 1H), 8.13 (s, 1H), 7.96-8.00 (m, 1H), 7.68-7.75 (m, 2H), 6.58 (d, *J* = 2.5 Hz, 1H), 6.27 (s, 1H)

**Preparation of (2-((7-chloroquinolin-4-yl)amino)phenyl)(morpholino)methanone, 56**

Methyl 2-((7-chloroquinolin-4-yl)amino)benzoate (0.6 mmol) was dissolved in THF (6 mL) and 1ml (0.1mmol) dispensed into a vial containing pre-weighed amines (0.15mmol). Triazabicyclo[4.4.0]dec-5-ene (0.03 mmol) was added to each vial, and the vials sealed and heated in a CEM Discover microwave reactor using initial power 150 W to 140 ^o^C for 10 minutes. The samples were dissolved in DMSO (0.5 mL), and purified by Mass Directed Auto Prep (Sunfire C18 column, MeCN/H_2_O with formic acid modifier), and the solvent removed under a stream of nitrogen to afford (2-((7-chloroquinolin-4-yl) amino)phenyl)(morpholino) methanone (0.7 mg, 2% yield).

**LCMS** (system A): t_ret_ 0.55 min, MH+ 368/370

**^1^H NMR** (400 MHz, CDCl_3_) δ ppm 8.63 (d, *J* = 5.5 Hz, 1H), 8.41-8.46 (m, 1H), 8.08 (d, *J* = 2.0 Hz, 1H), 7.94 (d, *J* = 8.6 Hz, 1H), 7.70 (d, *J* = 7.6 Hz, 1H), 7.43-7.53 (m, 2H), 7.34 (dd, *J* = 8.1, 1.5 Hz, 1H), 7.14-7.19 (m, 2H), 3.54-3.79 ppm (m, 8H)
